# Oxaloacetate anaplerosis differently contributes to pathogenicity in plant pathogenic fungi *Fusarium graminearum* and *F*. *oxysporum*

**DOI:** 10.1371/journal.ppat.1012544

**Published:** 2024-09-09

**Authors:** Soobin Shin, Seonghun Bong, Heeji Moon, Hosung Jeon, Hun Kim, Gyung Ja Choi, Do Yup Lee, Hokyoung Son

**Affiliations:** 1 Department of Agricultural Biotechnology, Seoul National University, Seoul, Republic of Korea; 2 Center for Eco-friendly New Materials, Korea Research Institute of Chemical Technology, Daejeon, Republic of Korea; 3 Research Institute of Agriculture and Life Sciences, Seoul National University, Seoul, Republic of Korea; Purdue University, UNITED STATES OF AMERICA

## Abstract

Anaplerosis refers to enzymatic reactions or pathways replenishing metabolic intermediates in the tricarboxylic acid (TCA) cycle. Pyruvate carboxylase (PYC) plays an important anaplerotic role by catalyzing pyruvate carboxylation, forming oxaloacetate. Although PYC orthologs are well conserved in prokaryotes and eukaryotes, their pathobiological functions in filamentous pathogenic fungi have yet to be fully understood. Here, we delve into the molecular functions of the ortholog gene *PYC1* in *Fusarium graminearum* and *F*. *oxysporum*, prominent fungal plant pathogens with distinct pathosystems, demonstrating variations in carbon metabolism for pathogenesis. Surprisingly, the *PYC1* deletion mutant of *F*. *oxysporum* exhibited pleiotropic defects in hyphal growth, conidiation, and virulence, unlike *F*. *graminearum*, where *PYC1* deletion did not significantly impact virulence. To further explore the species-specific effects of *PYC1* deletion on pathogenicity, we conducted comprehensive metabolic profiling. Despite shared metabolic changes, distinct reprogramming in central carbon and nitrogen metabolism was identified. Specifically, alpha-ketoglutarate, a key link between the TCA cycle and amino acid metabolism, showed significant down-regulation exclusively in the *PYC1* deletion mutant of *F*. *oxysporum*. The metabolic response associated with pathogenicity was notably characterized by S-methyl-5-thioadenosine and S-adenosyl-L-methionine. This research sheds light on how *PYC1*-mediated anaplerosis affects fungal metabolism and reveals species-specific variations, exemplified in *F*. *graminearum* and *F*. *oxysporum*.

## Author summary

Pyruvate carboxylase plays a crucial role in replenishing oxaloacetate in the tricarboxylic acid cycle, a fundamental carbon metabolic pathway. While PYC is present in both prokaryotes and eukaryotes, its specific contribution to the development and pathogenesis of fungal pathogens remains poorly understood. Our study focuses on elucidating the function of PYC, particularly investigating the effects of deleting the ortholog gene *PYC1* in two important fungal plant pathogens, *Fusarium graminearum* and *F*. *oxysporum*, which have distinct pathosystems. By conducting comprehensive genetic and metabolic profiling, we observed different impacts on fungal developmental processes and pathogenicity between the two species. In particular, the deletion of the *PYC1* gene in *F*. *oxysporum* led to a unique metabolic profile and the subsequent loss of pathogenicity. This research provides valuable insights into the distinct effects of *PYC1*-mediated anaplerosis on fungal metabolism in pathogenic fungi, contributing to a more profound understanding of the molecular mechanisms governing fungal pathogenesis.

## Introduction

The tricarboxylic acid (TCA) cycle, also known as the citric acid cycle or the Krebs cycle, is the central process in carbon metabolism. TCA cycle metabolites are critical for the functioning of the TCA cycle and are primarily considered essential precursors for cellular macromolecules such as nucleic acids, lipids, and proteins [1]. The TCA cycle comprises a series of reactions in a closed loop, with TCA-derived metabolites continually exiting the cycle to engage in various biochemical reactions. To maintain the balance of pathways that utilize TCA cycle metabolites, organisms replenish the pools of metabolic intermediates through a process known as anaplerosis [2]. The anaplerotic flux sustains the levels of TCA cycle metabolites to remain constant, thereby supporting cell growth and metabolism.

Pyruvate carboxylase (PYC, [EC 6.4.1.1]), has an important anaplerotic function by catalyzing the carboxylation of pyruvate to form oxaloacetate (OAA), a key intermediate in the TCA cycle involved in gluconeogenesis, lipogenesis, amino acid biosynthesis, the urea cycle, and the glyoxylate cycle [2, 3]. Therefore, the highly conserved PYC-encoding genes are implicated in diverse biological processes across organisms, including mammalian cells, fungi, and bacteria [4, 5]. PYC plays a vital role in sustaining cellular energy production by replenishing the TCA cycle and contributing to the metabolic reprogramming observed in cancer cells [6–9]. In human pathogenic bacteria, such as *Listeria monocytogenes*, PYC plays a crucial role in intracellular replication and virulence in host mammalian cells. [10] In yeast and filamentous fungi, mutations of PYC-encoding genes contribute differently to growth on glucose, affected by variations in the regulation of glyoxylate cycle activity. [11–14] These observations underscore the evolutionary conservation of the biochemical function of PYC while highlighting that each organism exhibits distinct carbon metabolic networks, leading to variations in underlying biological sub-processes.

The fungal genus *Fusarium* is one of the most important groups of plant-pathogenic fungi and impacts a wide variety of crops in agricultural regions worldwide [15]. *Fusarium graminearum* and *F*. *oxysporum* are regarded as major plant pathogenic fungi [16], and they are examples of fungi exhibiting distinct lifestyles and modes of interaction with their host plants. The infection process of *F*. *graminearum* is initiated by the deposition of the conidia or ascospores onto or inside the spike tissue. The fungus then spreads to aerial plant parts, resulting in head blight in cereal crops [17]. In contrast, *F*. *oxysporum* infects its host through root penetration, invades the cortex, colonizes the xylem vessels, and consequently induces wilt disease [18–20].

While PYC orthologs are conserved across prokaryotes and eukaryotes, the specific role of PYC-mediated anaplerosis in fungal development and pathogenesis remains largely unknown. This study aims to identify and compare the functions of the ortholog gene *PYC1* in two plant pathogenic fungi, *F*. *graminearum* and *F*. *oxysporum*. Our investigation revealed a crucial molecular function of *PYC1*, essential for virulence specifically in *F*. *oxysporum*. Comprehensive metabolomic profiling unveiled intricate metabolic functions linked to multiple factors such as pathogenicity, *PYC1* deletion, species, and incubation time. We identified a unique metabolic module and metabolites that may be metabolically associated with the exclusive loss of pathogenicity in the *F*. *oxysporum PYC1* deletion mutant. Additionally, *PYC1* in *F*. *oxysporum* was found to be necessary for host plant penetration and colonization, as well as for the utilization of pectin, a primary component of the plant cell wall. These findings, combined with the metabolomic analyses, offer valuable insights into the role of pyruvate carboxylation in fungal growth.

## Results

### Identification and characterization of the pyruvate carboxylase-encoding gene (*PYC1*) in *F*. *graminearum*

*PYC1* and *PYC2* genes, encoding the two isoenzymes of PYC were identified in the model organism *Saccharomyces cerevisiae* [21]. A BLASTp search was performed using *S*. *cerevisiae* Pyc1 as a query against the *F*. *graminearum* genome database. We identified a Pyc1 homolog in *F*. *graminearum* at the FGRAMPH1_01G23899 (formerly FGSG_07075) locus that encoded 1552 amino acids and was designated as *Fg*Pyc1. *Fg*Pyc1 shares 74% and 71% overall amino acid sequence identity with homologs in *S*. *cerevisiae* and *S*. *pombe*, respectively. Since *Fg*Pyc1 was annotated as a transcription factor in the previous study [22], we determined the exact sequence of *FgPYC1* using 3’-RACE-PCR analysis and confirmed that *Fg*Pyc1 does not contain any zinc finger motifs using the InterProScan search tool (S1A Fig). Phylogenetic analysis indicated that Pyc1 is highly conserved in prokaryotic and eukaryotic organisms and that *Fg*Pyc1 is closely related to the Pyc1 proteins of filamentous fungi (S1B Fig).

To investigate the biological functions of *FgPYC1* in *F*. *graminearum*, we disrupted the pyruvate carboxylase-encoding gene by replacing *FgPYC1* with a geneticin resistance gene cassette (*GEN*) in the *F*. *graminearum* wild-type strain Z-3639. The two deletion mutants were confirmed via Southern blotting hybridization (S2 Fig). Furthermore, the *FgPYC1* open reading frame (ORF) was fused to green fluorescent protein (GFP) under its native promoter and introduced into the Δ*Fgpyc1* mutant strain to produce two complementation strains (FgPYC1c). We comprehensively analyzed phenotypic changes in vegetative growth, conidiation, sexual reproduction, mycotoxin production, and virulence in the wild-type, deletion mutant, and complementation mutant strains.

**Fig 1:**
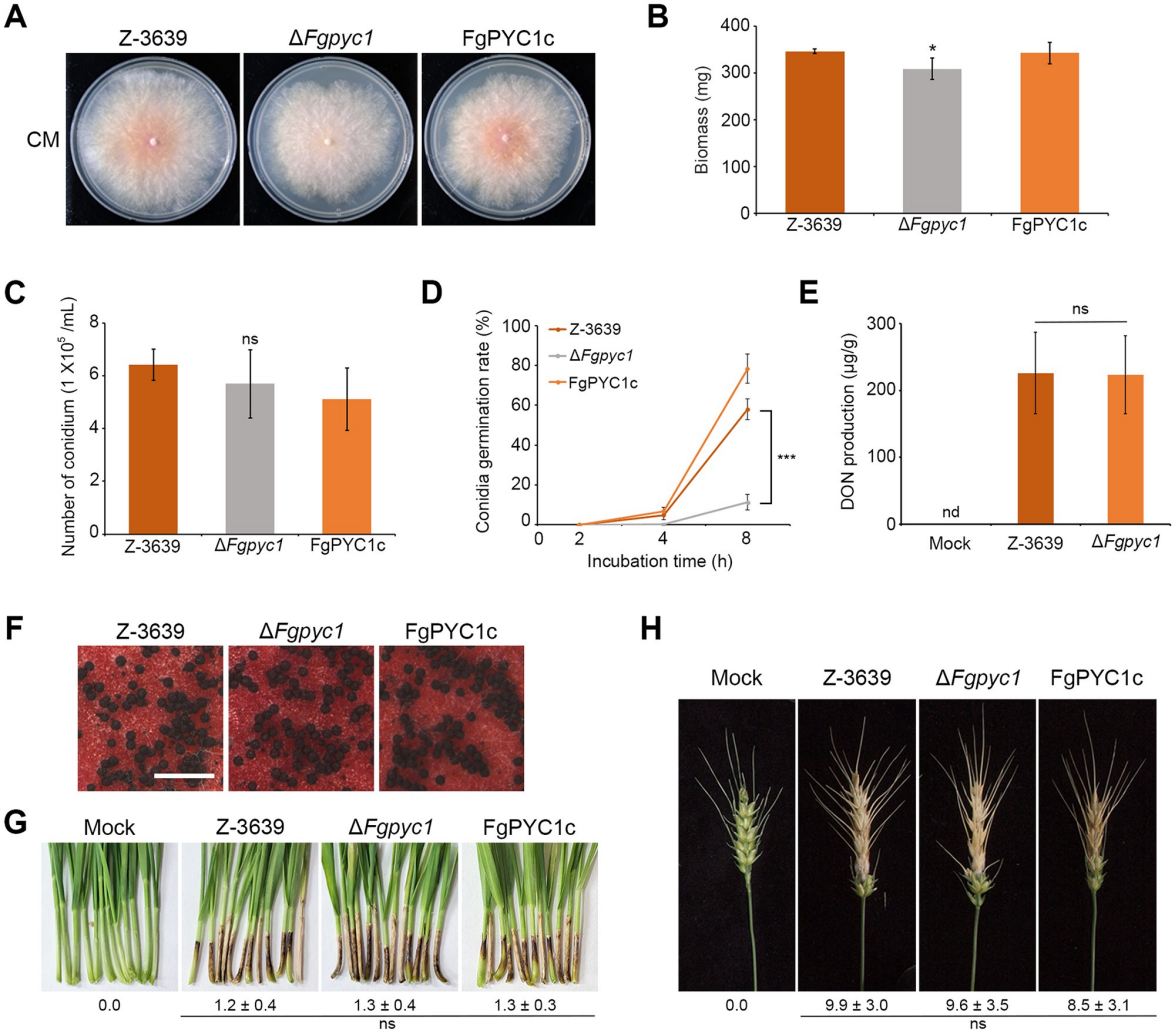
Phenotypical characterization of the Δ*Fgpyc1* mutant strains. A) Mycelial growth of *F*. *graminearum* strains on complete medium (CM). Pictures were taken 4 days after inoculation. B) Mycelial biomass (dry weight) of *F*. *graminearum* strains grown in CM. 1 × 10^6^ conidia of each strain were cultured on CM liquid medium for 5 days in a rotary shaker at 150 rpm, 25 °C. The mycelia were collected on filter paper and the dry weight of each strain was detected after the mycelium dried in a 55 °C baking oven for 48 hours. Error bars represent standard deviations of biological replicates (n = 3 for wild type, n = 6 for Δ*Fgpyc1* and FgPYC1c). C) Conidial production. The number of conidia generated by each strain was counted after 5 days of incubation in carboxymethyl cellulose (CMC) medium. Error bars represent standard deviations of biological replicates (n = 3 for wild type, n = 6 for Δ*Fgpyc1* and FgPYC1c). D) Conidia germination assay. The germination rates of 300 conidia in CM medium during 2, 4, and 8 hours (h) were measured for each strain. E) Deoxynivalenol (DON) production. A10 μL of conidial suspension (1 × 10^6^ conidia/mL) was injected into the center spikelet of a wheat head. Four to five spikes were inoculated per plant. The inoculated spikes were sampled at 5 dpi and ground and extracted with 84% acetonitrile. Reverse-phase HPLC with a C18 column was used for the analysis for DON detection. F) Perithecium formation. The perithecia were imaged 7 days after sexual induction on carrot agar. Scale bar = 1,000 μm. G) Virulence on wheat coleoptiles. Conidial suspension (10 μL, 1 × 10^6^ conidia/mL) obtained from each strain was inoculated into fresh wheat coleoptile wounds. The pictures were taken 5 days after inoculation. Mock inoculation was performed with distilled water. n = 12 and n = 12 independently inoculated spikelets for the wild-type and mutant strains, respectively. Lesion lengths were represented as mean ± SEM. ns; not significant. H) Virulence on wheat heads. The center spikelet of each wheat head was injected with 10 μL of conidial suspension. The pictures were taken 21 days after inoculation. Mock inoculation was performed with 0.01% Tween 20. The disease index (diseased spikelets per wheat head) is denoted below the picture. n = 15 and n = 15 independently inoculated spikelets for the wild-type and mutant strains, respectively. Two deletion mutant and two complementation strains were used in this experiment. Significant differences (*p < 0.05; **p < 0.01; ***p < 0.001), compared to the wild-type, are indicated by asterisks. ns: not significant.

On complete medium (CM), vegetative growth of the deletion mutants was slightly reduced compared to the wild type strain (Fig 1A and 1B). Conidial production was not significantly different in Δ*Fgpyc1* mutants compared to the wild-type strain (Fig 1C). In contrast, germination rates of conidia were significantly lower in Δ*Fgpyc1* mutants (11.2% at 8 hours) compared to the wild-type strain (58% at 8 hours) (Fig 1D). When evaluating mycotoxin production in flowering wheat, the quantities of deoxynivalenol (DON) and the expression levels of DON biosynthetic genes in the Δ*Fgpyc1* mutants were found to be comparable to those in the wild-type strain (Figs 1E and S3A). However, when DON level was investigated in the rice media, the amounts of DON was markedly reduced in Δ*Fgpyc1* mutants compared to the wild-type strain (S3B Fig). These data suggest that DON production may be influenced by the carbon content available to the fungus for growth and utilization. Additionally, since DON analysis in rice media requires a 4-week fungal culture, *FgPYC1* may have influenced specific metabolic process, such as DON biosynthesis, during this prolonged period, similar to other carbon metabolic enzymes reported earlier [23]. With respect to sexual development, Δ*Fgpyc1* mutants were not defective in perithecial maturation compared to the wild-type strain (Fig 1F). The virulence of each strain was evaluated through point inoculation on flowering wheat heads and coleoptile. The Δ*Fgpyc1* mutants were not defective in the infection of wheat coleoptiles and caused similar disease symptoms as the wild-type strain (Fig 1G). In wheat head infection experiments, the wild-type, deletion mutants, and complemented strains induced typical head blight symptoms within 21 days after inoculation (Fig 1H). Taken together, the result of the phenotypic analysis suggested that *PYC1*-mediated anaplerosis does not contribute significantly to virulence of *F*. *graminearum* on wheat.

### *FoPYC1* is important for vegetative growth, conidiation, and virulence in *F*. *oxysporum*

The genus *Fusarium* includes numerous plant pathogenic species with worldwide distribution, long recognized for their significance as plant pathogens. Given the differences in carbon metabolism associated with diverse lifestyles among *Fusarium* species, the biological function of *PYC1* may vary depending on the specific species.

**Fig 2:**
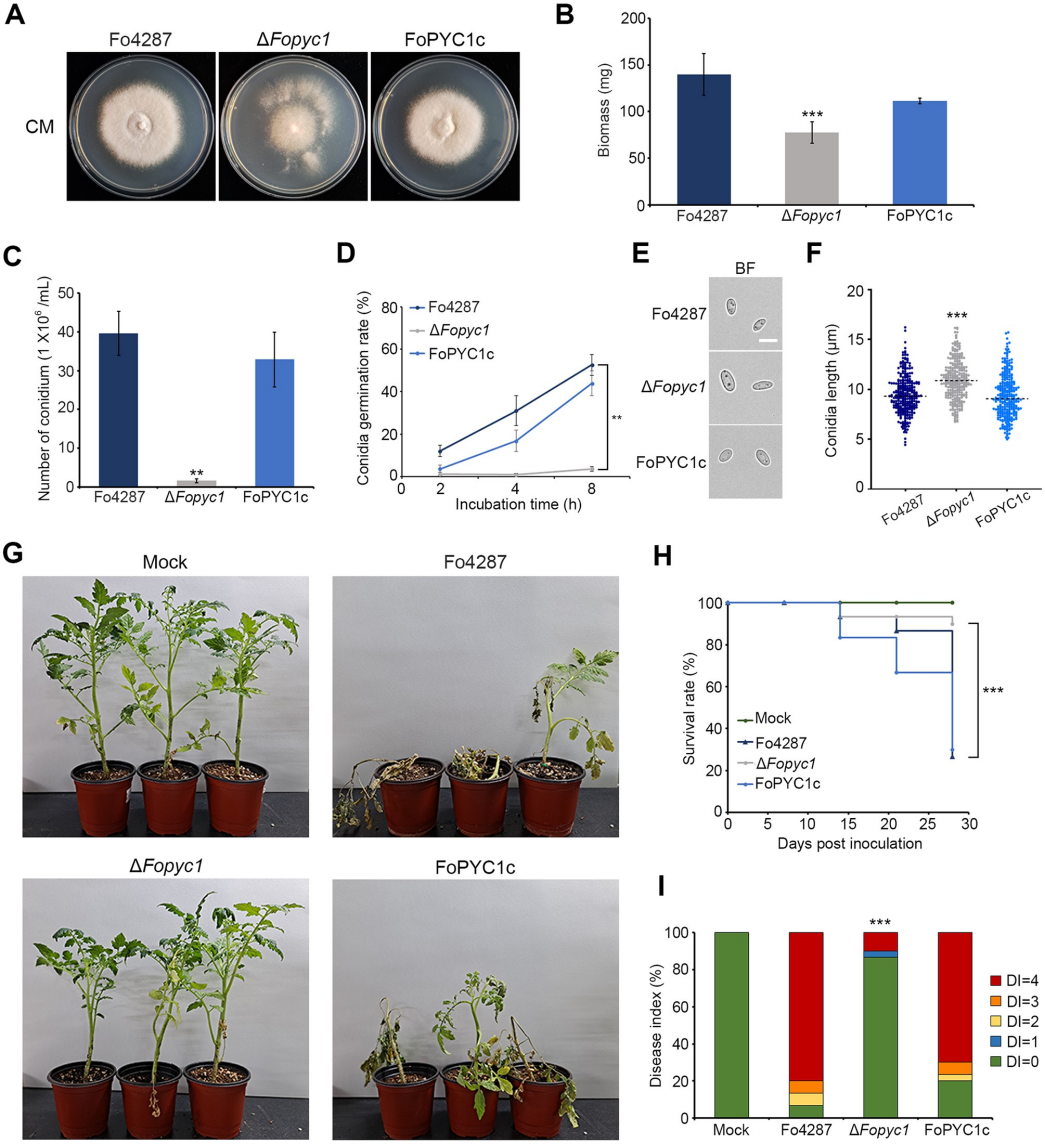
Phenotypical characterization and virulence of the Δ*Fopyc1* mutant strains. A) Mycelial growth of *F*. *oxysporum* strains on complete medium (CM). Pictures were taken 5 days after inoculation. B) Mycelial biomass (dry weight) of *F*. *oxysporum* strains grown in CM. 1 × 10^6^ conidia of each strain were cultured on CM liquid medium for 5 days in a rotary shaker at 150 rpm, 25 °C. The mycelia were collected on filter paper and the dry weight of each strain was detected after the mycelium dried in a 55 °C baking oven for 48 hours. Error bars represent standard deviations of biological replicates (n = 3 for wild type, n = 6 for Δ*Fopyc1* and FoPYC1c). C) Microconidia production. The number of microconidia generated by each strain was counted after 5 days of incubation in PDB medium. Error bars represent standard deviations of biological replicates (n = 3 for wild type, n = 6 for Δ*Fopyc1* and FoPYC1c). D) Conidia germination assay. The germination rates of 300 conidia in CM medium during 2, 4, and 8 h were measured for each strain. E) Microconidia morphology. Microconidia were induced in PDB medium for 5 days and subsequently observed by bright field (BF) microscopy. Scale bar: 10 μm. F) Dot plot showing the conidial length of each strain. The dashed line represents the median value. The length of 300 conidia induced on PDB was measured for each strain. ***p < 0.001. G, H, I) Fusarium wilt disease assay on tomatoes with *F*. *oxysporum* f. sp. *lycopersici* strain Fo4287. Two-week old seedlings were inoculated using a dipping protocol with 5 × 10^6^ microconidia mL^-1^ or water (mock inoculation). n = 15 and n = 15 independently inoculated seedlings for the wild-type and mutant strains, respectively. G) The pictures were taken 28 days after inoculation. H) Kaplan–Meier plot showing the survival of tomato plants. ***p < 0.001, versus the wild-type according to the log-rank test. I) Disease progress was assessed using a disease index. Disease index was scored on a scale of 0–4 [0, no symptoms; 1, slightly swollen and/or bent hypocotyl; 2, one or two brown vascular bundles in hypocotyl; 3, at least two brown vascular bundles and growth distortion (strong bending of the stem and asymmetric development); 4, all vascular bundles are brown, plant either dead. Two deletion mutant and two complementation strains were used in this experiment. Significant differences (*p < 0.05; **p < 0.01; ***p < 0.001), compared to the wild-type strain, are indicated by asterisks. ns: not significant.

In an attempt to identify the putative pyruvate carboxylase-encoding gene (*FoPYC1*) in *F*. *oxysporum* and elucidate its function in comparison to *F*. *graminearum*, we designated *Fo*Pyc1 as a Pyc1 ortholog in *F*. *oxysporum* f. sp. *lycopersici* at the FOXG_01733 locus, encoding 1197 amino acids. Similar to *F*. *graminearum*, *F*. *oxysporum* had only one Pyc1 ortholog, exhibiting a high amino acid sequence identity of 97% with *Fg*Pyc1. The disruption of the *FoPYC1* gene in *F*. *oxysporum* f. sp. *lycopersici* resulted in significant reductions in vegetative growth rates and altered colony morphology compared to the wild-type and complemented strains (Fig 2A and 2B). Moreover, conidial production in potato dextrose broth (PDB) medium was dramatically reduced in the Δ*Fopyc1* mutants compared to the wild-type strain (Fig 2C). Consistent with observations in *F*. *graminearum*, Δ*Fopyc1* mutants exhibited defects in conidia germination, with germination rates of 3.7% compared to the wild-type strain’s rate of 52.7% at 8 hours (Fig 2D). Furthermore, the deletion of *FoPYC1* led to the production of abnormal conidia, characterized by increased length and width compared to those of the wild-type strain (Fig 2E and 2F). To determine the role of *FoPYC1* in the virulence of *F*. *oxysporum*, the roots of 2-week-old tomato plants were inoculated with the *F*. *oxysporum* strains (Fig 2G–2I). Wilt symptoms and plant mortality were significantly reduced in plants inoculated with the Δ*Fopyc1* mutant, with 90% of the plants remaining alive after 28 days. The wild-type phenotype was fully restored in the complementation strain FoPYC1c. In addition, Δ*Fopyc1* · FgPYC1oe mutant strains successfully restored developmental defects in the *FoPYC1* deletion mutant (S4 Fig). Based on these results, the phenotypic differences resulting from *PYC1* deletion between *F*. *graminearum* and *F*. *oxysporum* are likely attributable to differences in carbon catabolism rather than sequence variations.

These findings indicate that *FoPYC1* plays key roles in vegetative growth and conidiation of *F*. *oxysporum*. Comparisons of phenotypes of *PYC1* mutants of *F*. *graminearum* and *F*. *oxysporum* indicate that the impact of *PYC1*-mediated anaplerosis differs in these two plant pathogenic fungi. Given that OAA serves as a carbon donor to replenish TCA cycle metabolites, our subsequent focus was to unravel the impact of OAA anaplerosis on fungal development and pathogenicity through metabolomics approaches.

### The deletion of *PYC1* depletes TCA cycle metabolites in *F*. *graminearum* and *F*. *oxysporum*

**Fig 3:**
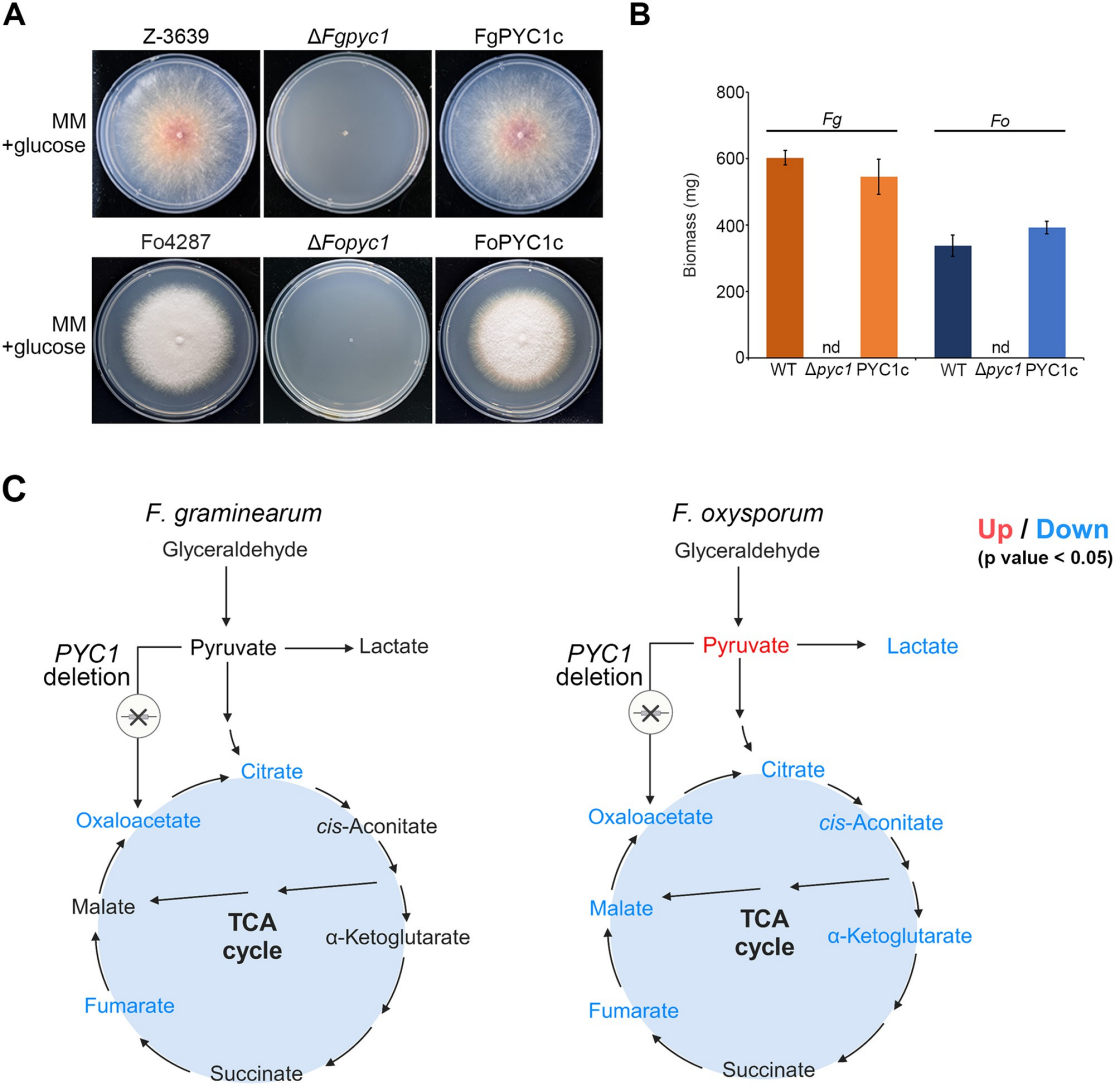
Effect of *PYC1* deletion on the growth of *F*. *graminearum* and *F*. *oxysporum* strains in a culture with glucose as a sole carbon source. A) Mycelial growth of *F*. *graminearum* and *F*. *oxysporum* strains on minimal medium (MM) with 2% glucose. Pictures were taken 5 days after inoculation. B) Mycelial biomass (dry weight) of *F*. *graminearum* and *F*. *oxysporum* strains in MM with glucose. 1 × 10^6^ conidia of each strain were cultured on MM liquid medium supplemented with 2% glucose (w/v) for 5 days in a rotary shaker at 150 rpm, 25 °C. The mycelia were collected on filter paper and the dry weight of each strain was detected after the mycelium dried in a 55 °C baking oven for 48 hours. Error bars represent standard deviations of biological replicates (n = 3 for wild type, n = 6 for Δ*pyc1* and PYC1c). nd: not detected. C) Overview of the metabolite profiles of mycelial samples of *F*. *graminearum* and *F*. *oxysporum* strains. TCA intermediates were detected by LC-Orbitrap MS; OAA and pyruvate were detected by LC-Orbitrap MS following 3-nitrophenylhydrazine derivatization (n = 6 for Z-3639, Fo4287, Δ*Fgpyc1*, and Δ*Fopyc1*). Fungal mycelium was pre-cultured in complete medium (CM) for 24 hours. Then, 25 mL of MM lacking a carbon source was transferred to a 100 mL flask. The pre-cultured mycelium was transferred to the MM, and the culture was incubated for 1 hour (subculture). After subculture, 2% glucose was added to the medium as a carbon source. The fungal cultures were then incubated for an additional 24 and 48 hours. Following incubation, the culture media were collected and used for metabolite profiling. One *PYC1* deletion mutant strain was used for metabolome analysis. p-value <0.05, Mann-Whitney.

As previously mentioned, the deletion of the Pyc1-encoding gene has been shown to abolish growth in glucose-supplemented medium in yeast species excluding the dimorphic yeast *Yarrowia lipolytica*, as well as the filamentous fungus *Aspergillus nidulans* [11–14]. Likewise, *PYC1* mutants of both *F*. *graminearum* and *F*. *oxysporum* were unable to grow in minimal medium (MM) with glucose as the sole carbon source (Fig 3A and 3B). The growth deficiency on glucose was restored when supplemented with aspartate, as previously reported in yeast strains (S5 Fig) [24]. However, when infected into tomato roots the presence of aspartate solution did not restore the virulence of Δ*Fopyc1* mutant strains (S6 Fig). In addition, direct supplementation of OAA to MM did not rescue the growth defect of the *PYC1* deletion mutants, as observed in human pathogenic bacteria [10]. These data indicate *F*. *graminearum* and *F*. *oxysporum* may not be able to directly uptake OAA due to the absence of oxaloacetate permease, as revealed by BLASTp analysis utilizing the *Arabidopsis thaliana* dicarboxylate transporter as a reference.

*PYC1*-mediated anaplerosis has been reported for its association with metabolic reprogramming in cancer cells [6, 7]. In terms of microorganisms, a previous study demonstrated that *PYC1*-mediated carboxylation of pyruvate is the predominant reaction leading to OAA in *L*. *monocytogenes* and *S*. *cerevisiae* [10, 25]. Therefore, we investigated whether *F*. *graminearum* and *F*. *oxysporum* also undergo significant metabolic alterations under *PYC1* deletion. In the presence of glucose as a sole carbon source, we examined metabolic differences between the wild-type and *PYC1* deletion mutant strains in *F*. *graminearum* and *F*. *oxysporum*. Firstly, targeted analysis was conducted for OAA and pyruvate measurement, applying derivatization with 3-nitrophenylhydrazine (3-NPH) to enhance sensitivity [26, 27]. OAA and pyruvate showed the characteristic peak profiles as previously reported, however, OAA was detected only one peak in low abundance. (S7–S9 Figs) [27]. In addition, untargeted metabolomics were employed to acquire a global metabolite profile, including TCA intermediates (e.g., citrate, cis-aconitate, alpha-ketoglutarate, succinate, fumarate, and malate) (Fig 3C and S1 Table). Most TCA cycle metabolites were down-regulated by *PYC1* deletion. Notably, *F*. *oxysporum* displayed higher levels of perturbation by *PYC1* deletion compared to *F*. *graminearum*. Additionally, Δ*Fopyc1* mutant strain exhibited higher levels of pyruvate and lactate compared to the wild-type strain, a pattern that was not seen in the Δ*Fgpyc1* mutant strain. This accumulation of pyruvate and lactate is likely a result of reduced activity caused by the significant decrease in TCA metabolites in the Δ*Fopyc1* mutant strain.

Targeted metabolic profiling revealed that *PYC1* deletion not only suppressed the conversion of pyruvate to OAA but also triggered reduction of the TCA cycle intermediates except succinate in *PYC1* deletion and *cis*-aconitate, alpha-ketoglutarate, and malate in the Δ*Fopyc1* mutant strains only. In summary, metabolome analysis unveiled that pyruvate carboxylase activity is the primary anaplerotic reaction for OAA synthesis in *F*. *graminearum and F*. *oxysporum*, exerting a significant impact on the TCA cycle.

### The *PYC1* deletion triggered a substantial impact on the global metabolome in both *F*. *graminearum* and *F*. *oxysporum*

**Fig 4:**
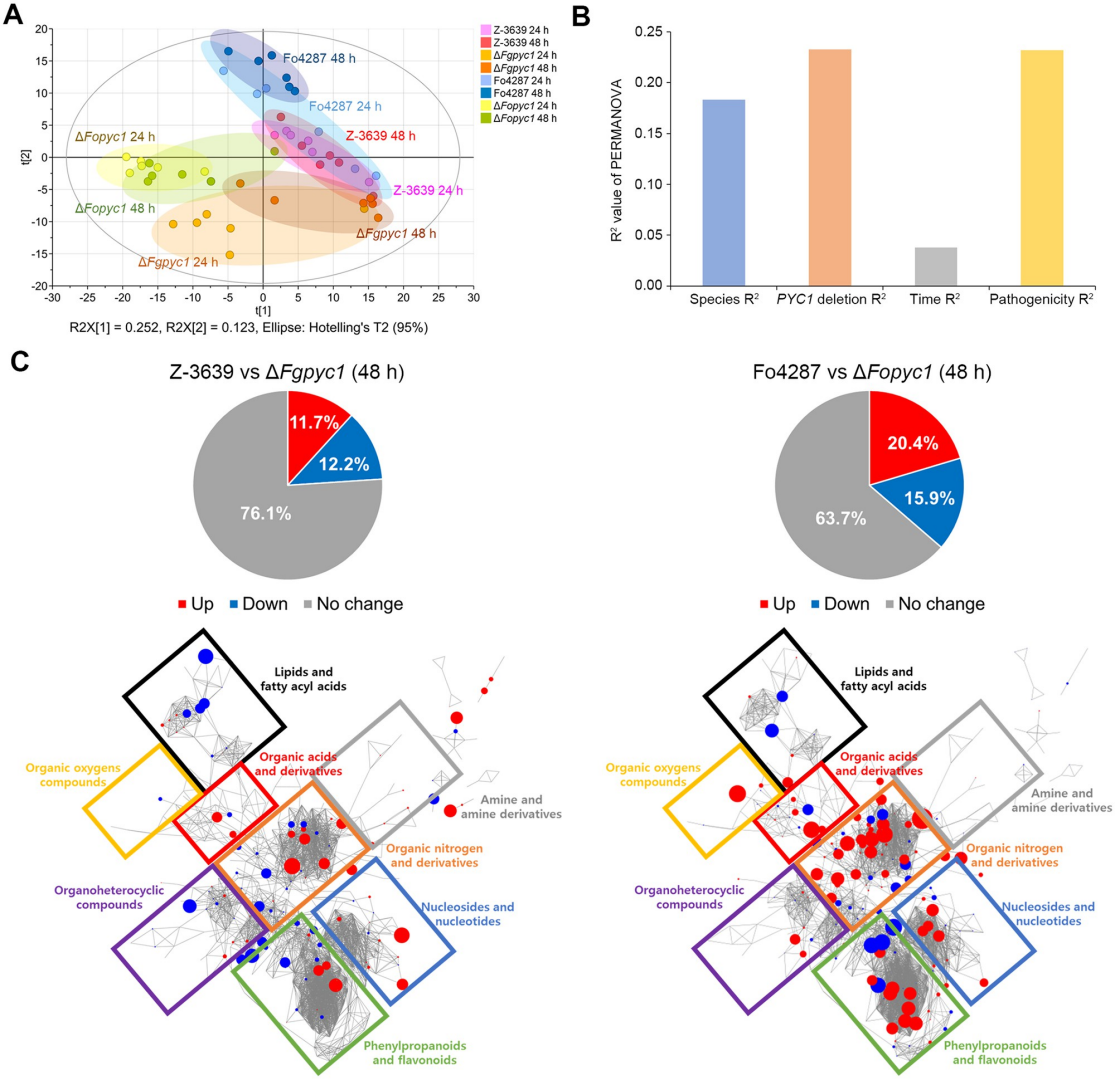
Metabolomic alteration of *PYC1* deletion on the *F*. *graminearum and F*. *oxysporum* strains. A) Unsupervised multivariate statistical analysis of integrative metabolic profiles based on principal component analysis (PCA). The metabolic profiles are separated along with the coordinated axis of species and *PYC1* deletion (n = 6) B) Permutational analysis of variance (PERMANOVA) for 4 factors (pathogenicity, *PYC1* deletion, species, and incubation time) (y axis: R square) C) Reconstructed metabolic network (48 hours culture). The metabolic network is reconstructed based on chemical similarity (Tanimoto score) and KEGG reaction pairs, which results in eight distinct clusters. Red and blue color represent the metabolites that are significantly up- and down-regulated in Δ*pyc1* mutants compared to the wild-type strains.

Next, we examined whether *PYC1* deletion had an impact on the global metabolic network through untargeted metabolomics. A total of 476 metabolites were detected in untargeted metabolomic profiles (S1 Table). Multivariate statistics were applied to capture characteristic profiles based on species, *PYC1* deletion (wild-type and mutant strains), pathogenicity, and incubation time. Abovementioned results, only *PYC1* deletion of *F*. *oxysporum* resulted in the loss of pathogenicity (Fig 2G–2I). Therefore, we considered Δ*Fopyc1* mutant strains as a weak pathogen and Z-3639, Δ*Fgpyc1* mutant, and Fo4287 as severe pathogens. The PCA score plot showed distinctive profiles according to different factors (R2X = 0.376 and Q2 = 0.117). The highest discrimination was observed for *PYC1* deletion along principal component 1, and for species along principal component 2. The profiles were not clearly separated by incubation time (Fig 4A). Permutational analysis of variance (PERMANOVA) was applied to quantitatively evaluate the explained variances by each aforementioned factor. The largest variance of the metabolome was determined by *PYC1* deletion (23.26%, p-value = 0.001), followed by pathogenicity (23.20%, p-value = 0.001) and species (18.33%, p-value = 0.001). Relative to the effect by *PYC1* deletion and species on the metabolome, the time effect appeared relatively small (3.80%, p-value = 0.141) (Fig 4B).

In addition, untargeted metabolic analysis revealed that the *PYC1* deletion affects the increase and decrease of metabolites directly related to fungal development. Both Δ*Fgpyc1* and Δ*Fopyc1* mutant strains over-produced potential toxic compounds, particularly indole derivatives, which coincided with a significant up-regulation of tryptophan-indole metabolism (S1 Table). Specifically, indole-3-lactic acid and N-acetyl tryptophan were found to be increased in both Δ*Fgpyc1* and Δ*Fopyc1* mutant strains, compared to the wild-type strain at 24 hours of incubations (Mann-Whitney p <0.05). Additionally, tryptophan, indole, 2-methyl indole, 3-formylindole, and indole-3-acetate were significantly increased in Δ*Fopyc1* mutants, compared to the wild-type strain in 24 hours of incubation. Compounds with indole ring have been reported to show fungal growth inhibition activity [28, 29]. In addition, a range of amino acids were significantly decreased, including glutamine, L-pyroglutamate, aspartate, and threonine in both Δ*Fgpyc1* and Δ*Fopyc1* mutant strains compared to the wild-type strain at 24 hours of incubation, which may lead to decreased conidia germination rate in these strains [30].

The *PYC1* deletion triggered marked perturbations in the global metabolome in both *F*. *graminearum* and *F*. *oxysporum* (Figs 4C and S10). In *F*. *graminearum* at 24 hours, 151 metabolites (31.7%) were impacted, with 93 metabolites enriched and 58 metabolites depleted in the Δ*Fgpyc1* mutant compared to the wild-type strain (S10 Fig). At the same time point, the Δ*Fopyc1* mutant exhibited significant upregulation of 185 metabolites, along with substantial downregulation of 33 metabolites compared to the wild-type strain (S10 Fig). At 48 hours, the Δ*Fgpyc1* mutant strains showed significant alterations in 114 metabolites (23.9%) compared to the wild-type strain (Fig 4C). Among these, 56 metabolites were enriched, while 58 were depleted in the Δ*Fgpyc1* compared to the wild-type strain. Δ*Fopyc1* mutant strains displayed significant changes in 173 metabolites (36.3%) compared to the wild-type strain, with 97 upregulated and 76 downregulated at 48 hours (Fig 4C).

We reconstructed a multi-layered metabolic network to systematically compare the effects of *PYC1* deletion on the global metabolome at 48 hours. Based on chemical structural similarity (Tanimoto score) and the KEGG reaction pair, organic nitrogen and derivative subnetwork especially highlighted the most dramatic discrepancy in *PYC1*-mediated anaplerosis between *F*. *graminearum* and *F*. *oxysporum*. The cluster of metabolic features retained the structural and biochemical association with an organic acids and derivative subnetwork, composed of TCA cycle intermediates directly affected by *PYC1* deletion. Additionally, the metabolic constituent unit of the secondary metabolite, phenylpropanoids-flavonoid subnetwork, exhibited up-regulation in Δ*Fopyc1* mutant compared to the wild-type strain, there is only minor metabolic alteration in Δ*Fgpyc1* mutant strain. These results collectively suggest that *PYC1* deletion affects the global metabolome differently in *F*. *graminearum* and *F*. *oxysporum*, with a more significant impact on *F*. *oxysporum*.

### *FoPYC1* contributes to penetration and root colonization

The successful penetration of living plant tissue by fungal pathogens encompasses a series of processes, including penetration, survival, symptom development, and invasive growth within the plant host [31]. Plant penetration is an energy-consuming process, as attaching spores to host surfaces and subsequent germination are known to require enormous metabolic energy [32, 33]. Based on metabolome analysis, we revealed that *PYC1* deletion resulted in a reduction of TCA cycle metabolites, consequently impairing energy metabolism. Anticipating a more pronounced defect in energy metabolism in Δ*Fopyc1* mutant compared to Δ*Fgpyc1* mutant strains, we hypothesized that this difference might affect energy-requiring steps in pathogenesis, such as plant penetration.

**Fig 5:**
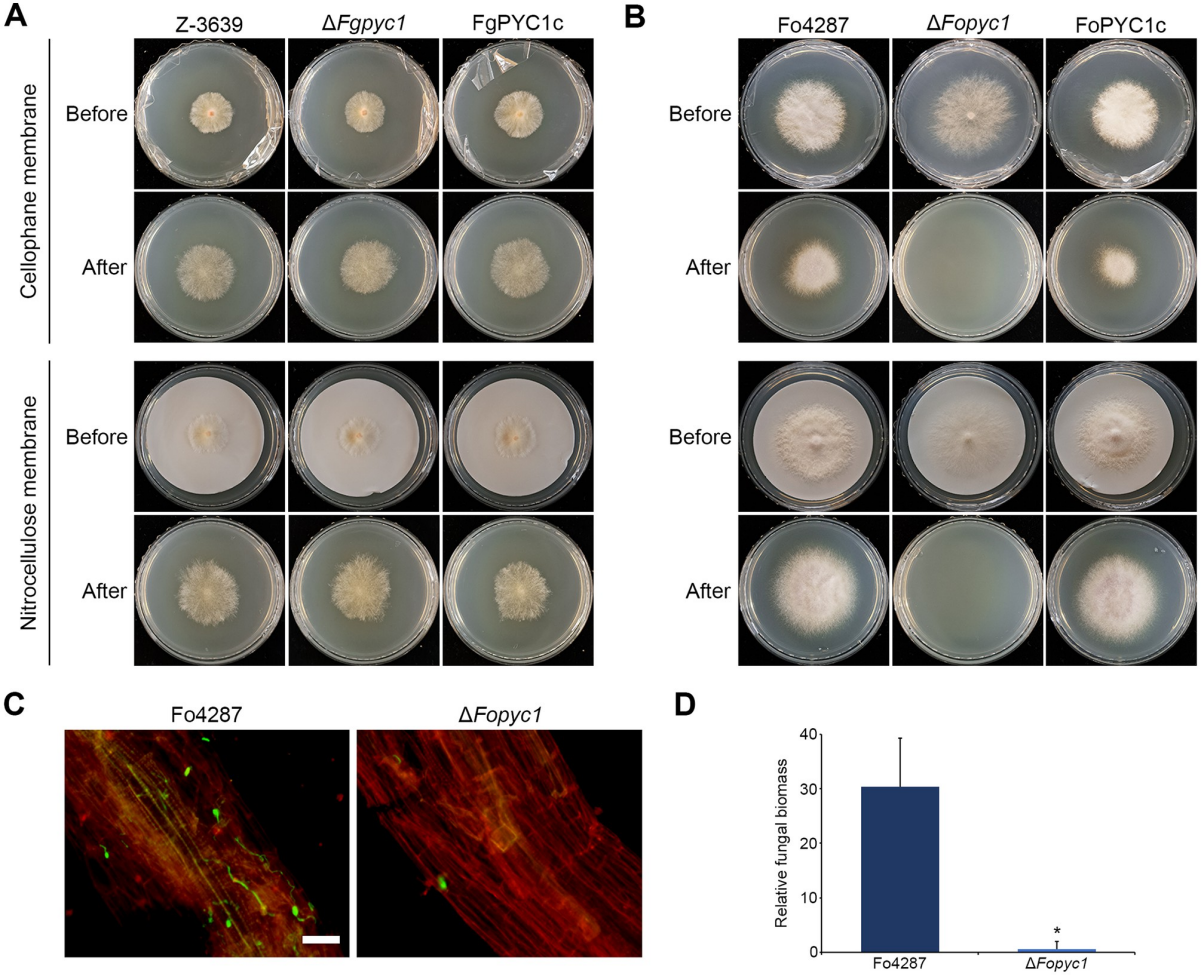
*FoPYC1* contribute to penetration, root colonization, and activation of cell wall degrading enzymes. A) *F*. *graminearum* and *F*. *oxysporum* strains grown on cellophane for 36 h and 72 h after strains had penetrated a cellophane membrane on the CM plate (Before). Following incubation, the membrane with the agar block on it was removed, and the plate was incubated for 2 days for readout. Pictures were taken 2 days after cellophane membranes were removed (After). ‘Before’ and ‘After’ refer to fungal culture plate with and without cellophane membrane. B) Fluorescence microscopy of tomato root colonization of *F*. *oxysporum* strains expressing pIGPAPA at 3 days post infection (dpi). Fungal fluorescence (green) is overlaid with the RFP filter of plant cell walls (red). Scale bars: 25 μm. C) Fungal biomass in the roots of tomato plants inoculated with the *F*. *oxysporum* strains was measured by qPCR of the Fo4287 specific genes (FOXG_16418) using total DNA extracted at 3 dpi. *F*. *oxysporum* DNA was calculated using the threshold cycle (ΔΔCt) method, normalized to the tomato *GADPH* gene. Error bars represent standard deviations of biological replicates (n = 3 for wild type, n = 6 for Δ*Fopyc1*). Two deletion mutant and two complementation strains were used in this experiment. Significant differences (*p < 0.05) compared to the wild-type are indicated by asterisks.

To investigate the energy-demanding factors affecting pathogenicity differences between Δ*Fgpyc1* and Δ*Fopyc1* mutants, we conducted a penetration assay. There were no discernible differences in penetration ability through cellophane and the challenging-to-degrade nitrocellulose membrane between the wild-type and Δ*Fgpyc1* mutant strains (Fig 5A). Interestingly, Δ*Fopyc1* mutants could not penetrate the cellophane or nitrocellulose membrane, suggesting that the failure of penetration may lead to a reduced virulence phenotype (Fig 5B). Moreover, fluorescence microscopy of tomato roots inoculated with the green fluorescent protein (GFP)-expressing Δ*Fopyc1* mutants confirmed a reduced ability to colonize tomato roots compared to the wild-type strain (Fig 5C). Quantitative PCR (qPCR) further verified that roots inoculated with Δ*Fopyc1* mutant strains contained less fungal biomass than those inoculated with the strain carrying the wild-type *PYC1* allele (Fig 5D). These findings indicate that Δ*Fopyc1* mutant strains failed to colonize and grow inside roots, resulting in reduced virulence.

### The metabolome was highly correlated with pathogenicity

**Fig 6:**
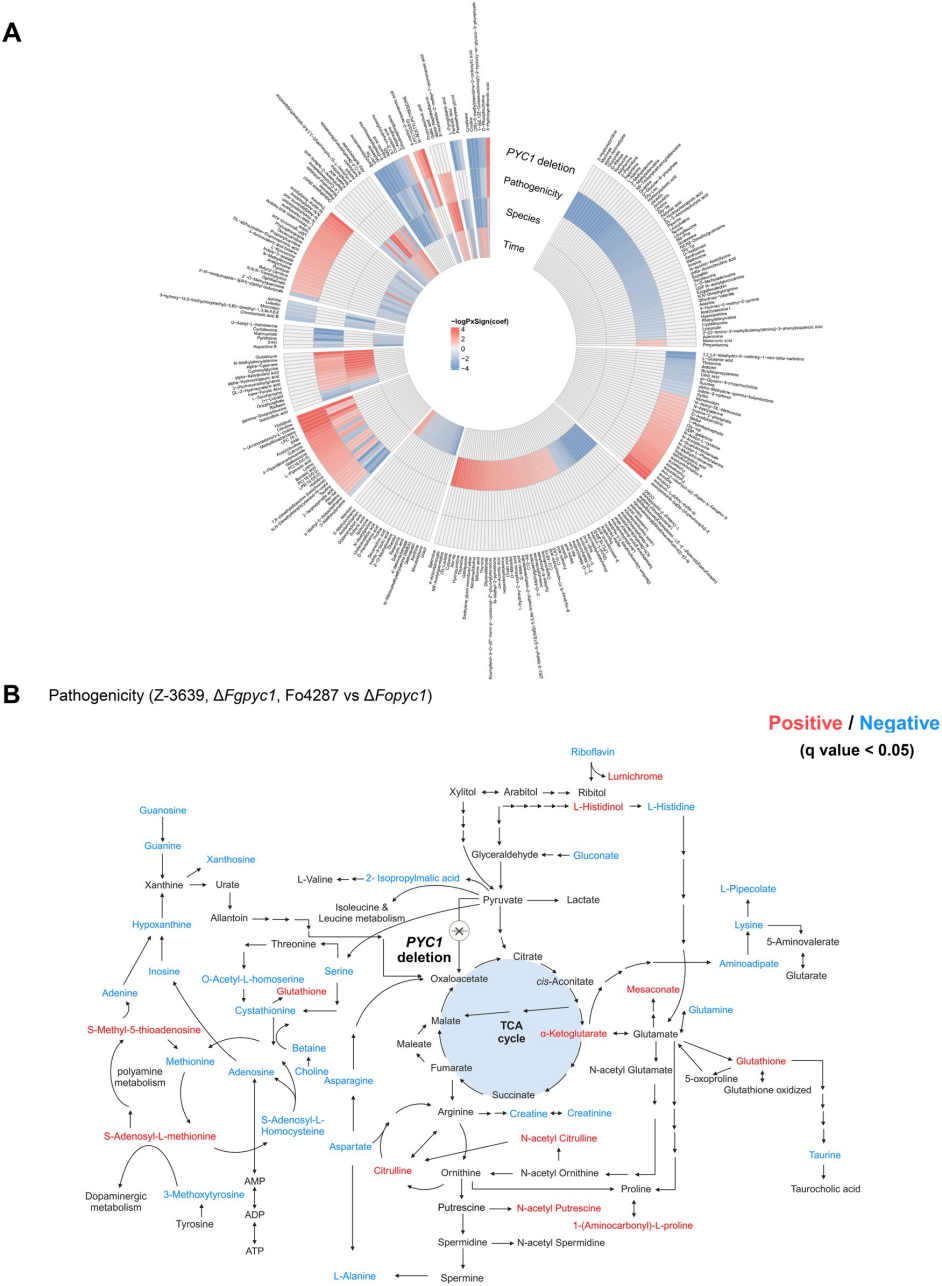
Comprehensive analysis of metabolic alteration through multiple linear regression. A) A circular heatmap showing metabolites that differ significantly between each factor (pathogenicity, *PYC1* deletion, species, and incubation time) (FDR <0.05, Benjamini-Hochberg correction). The four-outer layer heatmap indicates the correlation coefficient (–log (P value) x significance) using multiple linear regression model, where red/blue colors correspond to metabolites positive-/negative correlation. The four-inner circles of the heatmap represent the factor, from outer to inner: incubation time, species, *PYC1* deletion, and pathogenicity. Grey color indicates correlation with no factor. B) Metabolic pathway associated with pathogenicity of *F*. *graminearum* and *F*. *oxysporum* strains. Red and blue indicate significant positive and negative correlation coefficients, respectively (q < 0.05).

We characterized the metabolic features associated with each variation, associated with four variables: pathogenicity, species, *PYC1* deletion, and incubation time based on a multiple linear regression (MLR) analysis (Fig 6A and S2 Table). Among the 476 metabolites analyzed, 285 (59.9%) showed strong associations, as determined by the Benjamini–Hochberg false discovery rate (BH FDR) correction (q-value < 0.05). A total of 112 metabolites (23.5%) were associated with the *PYC1* deletion, of which 76 and 36 metabolites retained up-regulated and down-regulated in *PYC1* deletion, respectively. Species-associated metabolites were defined by 113 features (23.7%), while 80 metabolites (16.8%) underwent incubation time-dependent changes. The total number of metabolites altered according to pathogenicity is 125 (26.3%), 37 metabolites were positively correlated and 88 metabolites were negatively correlated with pathogenicity.

Metabolites associated with pathogenicity were mapped into an integrative pathway, focusing on the TCA cycle and amino acid metabolism using the KEGG mapper (Fig 6B). Notably, significant downregulation was observed in the majority of intermediates in methionine metabolism and purine metabolism. On the contrary, S-adenosyl methionine and S-methyl-5-thioadenosine exhibited a positive association with pathogenicity. Previously reported, these two metabolites were associated with polysaccharide degradation. In previous study, these metabolites were found to be essential for polysaccharide methylation in fungi and plants, contributing to the breakdown of polysaccharides, particularly pectin and polygalacturonic acid [34, 35].

### *FoPYC1* contributes to pectin utilization and activation of cell wall-degrading enzymes

Pectin is a major component of the plant cell wall, found in the middle lamella, primary and secondary walls [36]. Phytopathogens have evolved various cell wall-degrading enzymes (CWDEs) to penetrate cell wall carbohydrate barriers. Since the degraded cell wall components produced by CWDEs can enter the TCA cycle, *FoPYC1* could potentially impact the utilization of plant-derived carbon sources such as pectin. Consequently, we aimed to investigate whether *PYC1* is required for pectin utilization affecting vegetative growth and the activation of CWDEs in the presence of pectin.

**Fig 7:**
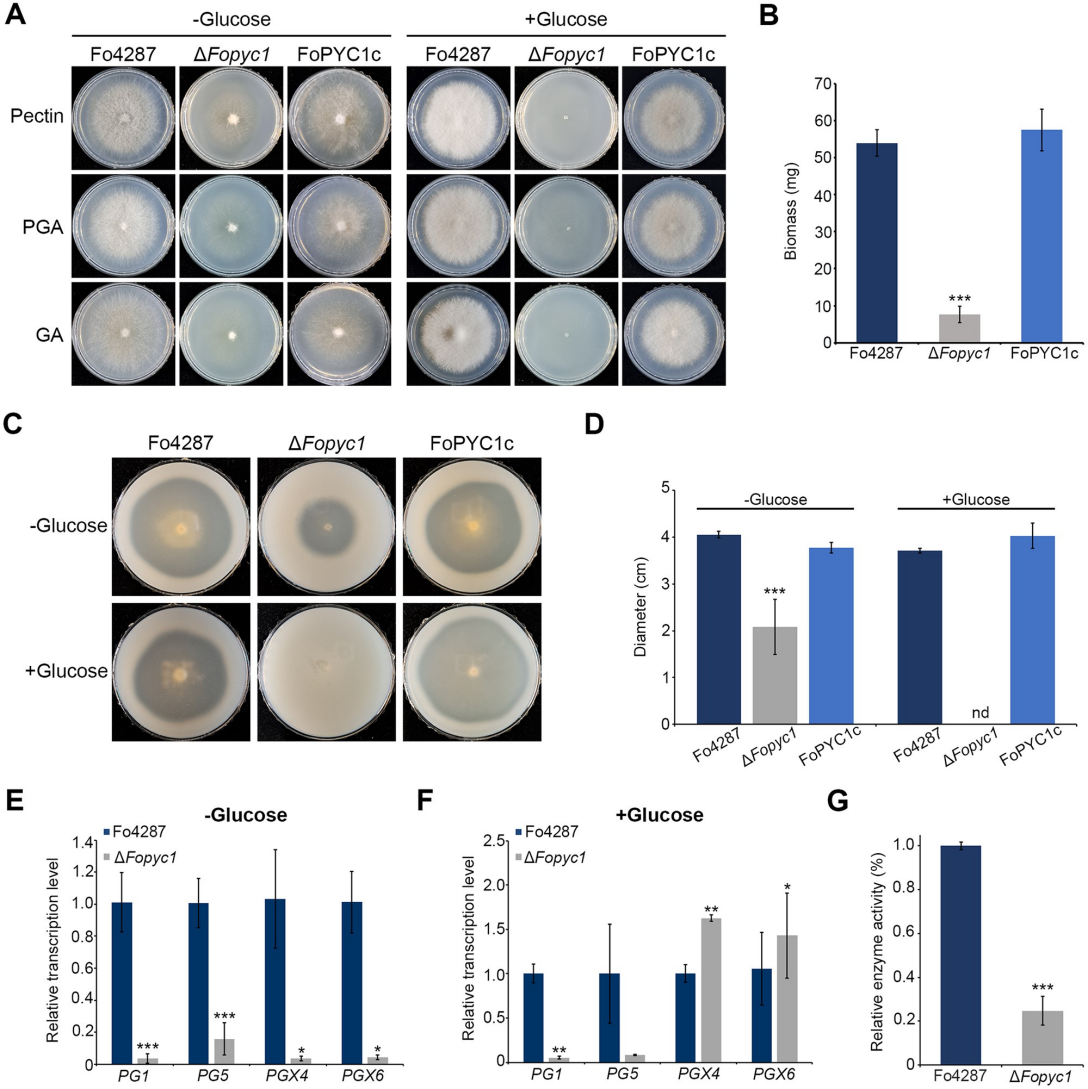
Reduced pectin utilization of Δ*Fopyc1* mutant strains. A) Mycelial growth of *F*. *oxysporum* strains on MM with 0.5% pectin, polygalacturonic acid (PGA), and galacturonic acid (GA) and supplemented with glucose. Pictures were taken 3 days after inoculation. B) Mycelial biomass (dry weight) of *F*. *oxysporum* strains grown in MM with 0.5% pectin. 1 × 10^6^ conidia of each strain were cultured on MM liquid medium with 0.5% pectin for 5 days in a rotary shaker at 150 rpm, 25 °C. The mycelia were collected on filter paper and the dry weight of each strain was detected after the mycelium dried in a 55 °C baking oven for 48 hours. Error bars represent standard deviations of biological replicates (n = 3 for the wild-type, n = 6 for Δ*Fopyc1* and FoPYC1c). C) Polygalacturonase plate assay showing halo zones indicating the PGA hydrolysis. Effect of 2% glucose supplementation to plate was also investigated. D) The halo diameter on each of the plates was measured. Error bars represent standard deviations of biological replicates (n = 3 for the wild-type, n = 6 for Δ*Fopyc1* and FoPYC1c). nd: not detected. E, F) Transcript levels of PG-encoding genes in *F*. *oxysporum* strains relative to actin were measured by quantitative reverse transcription-polymerase chain reaction (qRT-PCR). Mycelia were transferred to MM with E) 0.5% pectin and F) 0.5% pectin supplemented with 2% glucose and incubated for 4 h. The relative transcript abundances of the indicated gene in Fo4287 were arbitrarily set to 1. G) Total extracellular PG activity from culture supernatants in the *F*. *oxysporum* strains after 6 h of growth in pectin, using the Nelson–Somogyi method. Two deletion mutant and two complementation strains were used in this experiment. Significant differences (*p < 0.05; **p < 0.01; ***p < 0.001) in the relative transcript level of each gene in comparison with the wild-type strain are indicated with asterisks.

Supporting this hypothesis, the Δ*Fopyc1* mutant strains exhibited reduced radial growth on MM supplemented with D-galacturonic acid (GA), polygalacturonic acid (PGA), and pectin (Fig 7A). The biomass of Δ*Fopyc1* mutant strains cultured in MM supplemented with pectin was also significantly reduced, suggesting that *FoPYC1* is crucial for pectin metabolism (Fig 7B). In addition, polygalacturonase (PG) activities on PGA were analyzed to assess the effect of *PYC1* deletion on the secretion of pectinolytic enzymes and significant differences between the wild-type and Δ*Fopyc1* mutant strains were observed (Fig 7C and 7D). Additionally, Δ*Fopyc1* mutant strains were unable to grow in a medium supplemented with glucose, despite the presence of alternative carbon sources like pectin, PGA, and GA (Fig 7A–7D). This may be attributed to the toxic compounds produced by the Δ*Fopyc1* mutant strains, as described above (S1 Table). We also measured the expression of previously reported PG-encoding genes (*PG1*, *PG5*, *PGX4*, and *PGX6*) of *F*. *oxysporum* [37]. Compared to the wild-type strain, all PG-encoding genes of Δ*Fopyc1* mutants showed significantly lower transcript levels in the presence of pectin (Fig 7E). Interestingly, in the presence of pectin and glucose, we observed a decrease in the expression of endoPG genes (*PG1* and *PG5*) in the Δ*Fopyc1* mutants, while the expression of exoPG (*PGX4* and *PGX 6*) increased (Fig 7F). These results suggest that carbon catabolite repression by glucose might affect endoPG and exoPG in different ways when *PYC1* is deleted. Regarding the expression of the major CWDEs genes, we measured the total extracellular PG activity from the culture supernatant of the wild-type and Δ*Fopyc1* mutant strains. We found about a five-fold lower induction of enzymatic activity in Δ*Fopyc1* mutants compared to the wild-type strain (Fig 7G). Collectively, these experiments indicate that *PYC1* deletion mutants exhibit impairments in pectin utilization as well as in the utilization of primary carbon sources such as glucose.

## Discussion

Fungi exhibit a remarkable capacity to utilize diverse carbon sources, necessitating metabolic reprogramming in response to changes in substrate availability [38]. Particularly during infection, pathogenic fungi face host systems, prompting genetic mechanisms that modulate their growth and development to ensure successful survival and proliferation within the host [39]. Given its central position in carbon metabolism, the TCA cycle plays a pivotal role in orchestrating global gene expression and various cellular responses, thereby supporting pathogenesis in pathogenic fungi [40, 41]. Additionally, various carbon pathways supporting the TCA cycle affect downstream processes such as energy metabolism and biosynthetic pathways.

Previous studies have demonstrated that specific carbon metabolism genes play a role in fungal development and virulence in plant pathogenic fungi [39]. Notably, isocitrate lyase (ICL1), a key enzyme in the glyoxylate cycle, showed elevated expression during host infection in the rice blast pathogen *Magnaporthe oryzae* and was required for full virulence in both *M*. *oryzae* and *F*. *graminearum* [42, 43]. Deletion of the ATP citrate lyase-encoding gene resulted in defects in sexual development and virulence in *F*. *graminearum* [44], while acetyl coenzyme A synthetase and pyruvate decarboxylase have crucial roles in perithecium development and maturation in *F*. *graminearum* [45, 46]. Disruption of the malate dehydrogenase-encoding gene in *F*. *oxysporum* led to decreased penetration ability and delayed development of disease symptoms [47].

Given that anaplerosis serves as a key carbon donor replenishing the TCA cycle, it has the potential to influence multiple developmental processes in plant pathogenic fungi. This study focuses on characterizing the molecular functions of *PYC1* in *F*. *graminearum* and *F*. *oxysporum*, which might employ carbon metabolism differently during pathogenesis. Especially, as observed in the conidia germination assay in both fungi, the negative impact of *PYC1* deletion on germination of conidia in both *F*. *graminearum* and *F*. *oxysporum* suggests that *PYC1* has a significant impact on some energy-requiring processes [48, 49].

A noteworthy observation is the differential outcomes of *PYC1* deletion on fungal development between *F*. *graminearum* and *F*. *oxysporum*. *PYC1* deletion led to more severe developmental defects in *F*. *oxysporum* compared to *F*. *graminearum*, highlighting its greater importance for virulence in *F*. *oxysporum*. Our findings suggest that the role of *PYC1* in the TCA cycle can impact processes downstream of the cycle that are crucial for pathogenicity of *F*. *oxysporum*.

Metabolome analysis demonstrated that the impact of the *PYC1* deletion extended beyond OAA levels, reaching into the TCA cycle. The resulting changes in central carbon metabolism propagated to global metabolism. Utilizing targeted and untargeted metabolomic profiling, we primarily identified a substantial depletion of OAA due to *PYC1* deletion in both species. Notably, even with glucose feeding, OAA levels were not restored, implying that these mutants failed to meet the biosynthetic demands of the TCA cycle. Taken together, these results provide clear evidence that Pyc1 activity serves as the primary anaplerotic reaction for OAA synthesis in *Fusarium* spp.

The deletion of *PYC1* gene and the subsequent loss of pathogenicity are metabolically interconnected. Based on the results of the permutational analysis of variance, the highest levels of metabolic variance were explained by *PYC1* deletion and pathogenicity, surpassing other factors (e.g., incubation time and species). In particular, pathogenicity was characterized by enriched organic nitrogen and derivatives, including amino acids. Several amino acids exhibited significant changes associated with pathogenicity in our experimental setting. These alterations coincided with changes in alpha-ketoglutarate, a key intermediate connecting the TCA cycle with amino acid metabolism.

We identified a key metabolic module directly associated with pathogenicity. Within the module, the levels of methionine and purine metabolism were negatively correlated with pathogenicity, excluding S-adenosyl methionine and S-methyl-5-thioadenosine. Previous studies have established the essential role of these metabolites in polysaccharide methylation, facilitating hydrolysis [50, 51]. Specifically, in pectin metabolism, S-adenosyl methionine played a critical role in the methylation of polygalacturonic acid, a major component of pectin [52, 53]. Notably, given that pectin is the most abundant polysaccharide in plant cell walls and the regulation of pectinolytic enzymes is related to fungal pathogenicity [54], the down-regulation of S-adenosyl methionine and S-methyl-5-thioadenosine during the initial penetration phase may result in a loss of penetration ability.

As expected, *PYC1* contributes to the utilization of secondary carbon sources such as pectin. In addition, the activation of CWDEs in response to pectin was significantly reduced, leading to a decrease in total extracellular PG activity in Δ*Fopyc1* mutants. Since CWDEs have been regarded as virulence factors in *F*. *oxysporum* [37], the impaired expression of endoPG and exoPG in *PYC1* mutants may impair pathogenesis. Taken together, these findings suggest that the *PYC1* deletion can lead to the failure of host penetration, impaired utilization of pectin, and ultimately reduced fungal proliferation within the host plant.

**Fig 8:**
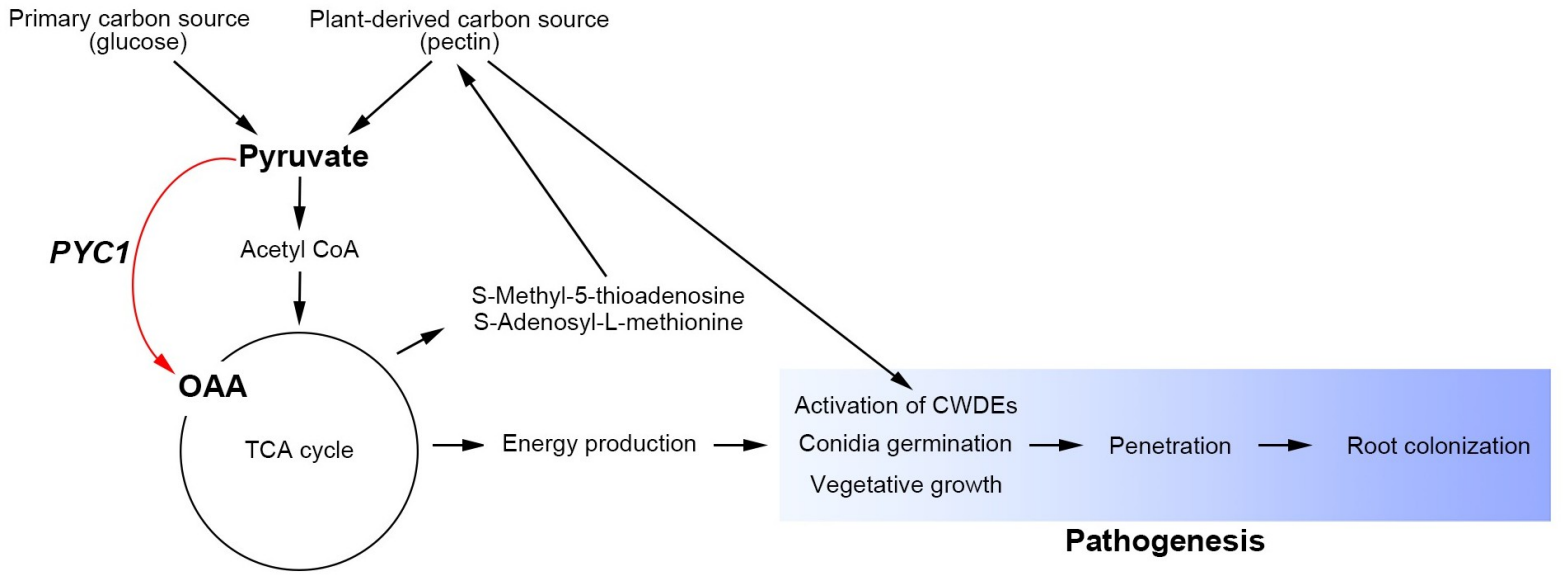
Proposed model of *PYC1* contribution in plant pathogenic fungi.

In conclusion, our study uncovers the previously uncharacterized role of *PYC1* in the two plant pathogenic fungi. We revealed that anaplerosis dependency on fungal development is higher in *F*. *oxysporum* than in *F*. *graminearum*, resulting in differences in virulence. This hypothesis is supported by the metabolomics approaches of the *PYC1* mutants, where global metabolic alterations are induced by a single *PYC1* gene, and key metabolic modules directly related to pathogenicity were identified (Fig 8). While the precise modes of action of *PYC1* remain to be elucidated, our results suggest that OAA anaplerosis differently contributes to fungal development and ultimately virulence in plant pathogenic fungi.

## Material and methods

### Fungal strains, plant cultivar, and culture conditions

The *F*. *graminearum* wild-type strain Z-3639 and the *F*. *oxysporum* f.sp. *lycopersici* wild-type strain 4287 (Fo4287) were used as parental strains for transformation experiments. All strains were stored as mycelial suspensions in a 20% glycerol solution at -80 °C. The *FgPYC1* deletion mutants used in this study have been constructed previously [22]. The culture media were prepared following the *Fusarium* laboratory manual [55]. Conidial production was induced in carboxymethyl cellulose (CMC) medium for *F*. *graminearum*, and potato dextrose broth (PDB) for *F*. *oxysporum* [56]. The growth temperature for fungal strains was set at 25°C.

For the wheat coleoptile assay, the wheat cultivar Eunpamil was used in this study and cultivated in a growth chamber at 20 °C/ 20 °C (day / night cycle) with 70% relative humidity. For the wheat head assay, the wheat cultivar Eunpamil was used in this study and cultivated in a green house. For the tomato root infection assay, the tomato cultivar Hongilpoom was used in this study and cultivated in soil, in a growth chamber 25 °C/ 25 °C (day / night cycle) with 70% relative humidity.

### Vegetative growth, conidiation, and sexual development

Colony morphology was assessed on CM and MM 4–5 days after inoculation. For conidiation assays, five fresh mycelial plugs from CM were inoculated in 50 mL of CMC for *F*. *graminearum* and PDB for *F*. *oxysporum* for 5 days on a rotary shaker (150 rpm) and the number of conidia was measured with a hemocytometer. The structure of spores was observed under a light microscope at 400 × magnification (Leica DM500, Jena, Germany). The conidial lengths were examined based on measurements from 100 spores per isolate using ImageJ [57] and the dot plots were drawn with GraphPad Prism 8.

For sexual development of *F*. *graminearum*, fungal strains were grown on carrot agar for 7 days, and aerial mycelia were removed with 0.4 mL of a sterile 2.5% Tween 60 solution [55]. The plates were then incubated under near-UV light (wavelength: 365 nm; Sankyo Denki, Tokyo, Japan) for 7 days.

For biomass quantification, 1 × 10^6^ conidia of each strain were cultured on CM or MM liquid medium for 5 days in a rotary shaker at 150 rpm, 25 °C. The mycelia were collected on filter paper and the dry weight of each strain was detected after the mycelium dried in a 55 °C baking oven for 48 hours.

Pectin utilization plate assay of *F*. *oxysporum* strains was performed on agar plates containing minimal (MM) medium, 1.5% bacto agar (Difco, Detroit, USA) and one of the following substrates: 0.5% (w/v) pectin from citrus fruit (Sigma-Aldrich, Saint Louis, USA), 0.5% (w/v) PGA, 0.5% (w/v) GA, 2% (w/v) glucose or a combination as specified.

For cell wall degrading enzyme induction, 5 × 10^6^ *Fusarium* microconidia were grown in PDB medium for 24 hours. The mycelia were collected through filter paper, washed with distilled water, transferred to flasks containing MM supplemented with 0.5% (w/v) pectin from citrus fruit and harvested within the indicated time period.

### Nucleic acid manipulations, Southern blotting and PCR

The genomic DNA was extracted from freeze-dried mycelia powder according to the *Fusarium* laboratory manual [55]. Total RNA was extracted from mycelia ground in liquid nitrogen using the Easy-Spin Total RNA Extraction Kit (Intron Biotech, Seongnam, Republic of Korea). Standard protocols were used for restriction endonuclease digestion and agarose gel electrophoresis [58]. Southern blot hybridization was performed using the North2South Biotin Random Prime Labeling Kit and the North2South Chemiluminescent Hybridization and Detection Kit (Thermo Scientific, USA). The PCR primers (S3 Table) were synthesized by an oligonucleotide synthesis facility (Bioneer, Seoul, Republic of Korea).

### Rapid amplification of cDNA ends (RACE)-PCR

We determined the *FgPYC1* open reading frame (ORF) by 5′/3′ rapid amplification of cDNA ends (RACE) PCR using the SMARTer RACE 5′/3′ kit (Takara Shuzo, Japan) following the manufacturer’s instructions. 3’-RACE PCR products were cloned into a pRACE vector and then directly sequenced.

### Genetic manipulations and fungal transformations

The double-joint (DJ) PCR method was used to generate the fusion PCR products required for gene deletion and complementation [59]. Fungal transformation was performed as previously described [22].

To construct deletion mutants, the 5’ and 3’ flanking regions of the target genes were amplified from the genomic DNA, and the geneticin resistance gene cassette (*GEN*) was amplified from pII99 [60]. Three fragments were fused by the DJ PCR method, and the final constructs were amplified using the nested primers. The resulting amplicons were transformed into the fungal wild-type protoplasts and the mutants were confirmed by Southern hybridization with a flanking region probe.

To complement the Δ*Fgpyc1* mutant, the ORF of *Fg*Pyc1 and its own promoter were amplified and fused with green fluorescent protein and the hygromycin resistance cassette (*HYG*) amplified from pIGPAPA vector [61]. The fusion construct was introduced into the *FgPYC1* deletion mutant and complementation strains were confirmed by Southern hybridization.

For complementation of the Δ*Fopyc1* mutant, the *FoPYC1*-*GFP* fusion construct was generated via yeast gap repair approach [62]. The ORF of *Fo*Pyc1 and its native promoter were amplified from the genomic DNA of the wild-type strain. The resulting construct and Xho1-digested pDL2 were co-transformed into the yeast strain PJ69-4A [63] using the Alkali-Cation Yeast Transformation Kit (MP Bio, Seoul, Republic of Korea). The *FoPYC1*-*GFP* fusion vector obtained from the yeast transformants was transformed into *Escherichia coli* DH10B. After verification by sequencing, the plasmid DNA was extracted with the DNA-spin Plasmid DNA Purification Kit (Intron Biotech, Seongnam, Republic of Korea) and used to transform the Δ*Fopyc1* mutant strain.

For investigating the interchangeability of *PYC1* between *F*. *graminearum* and *F*. *oxysporum*, the *RP27*-*FgPYC1-GFP* fusion construct was also generated via yeast gap repair approach. After verification by sequencing, the plasmid DNA was used to transform the Δ*Fopyc1* mutant strain.

To generate the cytosolic GFP-expressed mutant, pIGPAPA vector was introduced to the wild-type Fo4287 and Δ*Fopyc1* mutant strains, respectively. Fungal transformants showing the brightest fluorescence were used in subsequent microscopy analysis.

### Microscopic observation

Microscopic observation was performed with a Leica DM6 B microscope (Leica Microsystems, Wetzlar, Germany) that was equipped with a Leica DMC6200 camera using the fluorescent filter GFP (part no. 11504166). The perithecia were imaged 7 days after sexual induction using a Zeiss SteREO Lumar.V12 microscope (Carl Zeiss, Germany).

### Conidia germination assay

Investigation of conidia germination rate was performed as previously described with minor modifications [64]. The assay was performed in sterile 24-well plates (SPL Life science, Pocheon, Republic of Korea). A conidia suspension (10^7^ spores) was added to liquid CM (1 mL) and incubated on a rotary shaker (150 rpm) for 2 hours, 4 hours, and 8 hours. Conidial germination was determined microscopically by counting 100 conidia/ well and a microconidium was considered germinated if the germ tube length was longer than or equal to the spore [65]. The experiments were performed with three replicates each time.

### Virulence test

The point inoculation method of wheat heads with *F*. *graminearum* was performed to assay fungal virulence as previously described [44]. Conidia were harvested from CMC cultures and 10 μL of each suspension (10^5^ conidia/mL) was injected into the center spikelet of a wheat head. The inoculated wheat plants were incubated in a humid chamber for 3 days and grown in a greenhouse for an additional 18 days.

For wheat coleoptile virulence assay, two-day-old seedlings of wheat were used for the coleoptile infection assay according to previous study with modifications [66]. Two days after seed sowing, the top 2 to 3 mm of the coleoptiles were removed, and conidial suspension (10 μL, 1 × 10^6^ conidia/mL) obtained from each strain was inoculated to fresh wheat coleoptile wounds. Distilled water-inoculated coleoptiles with water served as a mock control. After inoculation, the seedlings were grown in a growth chamber at 25 °C and 95% humidity.

Tomato root infection assays with *F*. *oxysporum* were performed as previously described on 2-week old seedlings using a dipping protocol with 5 × 10^6^ microconidia mL^−1^ [67]. The inoculated tomato plants were incubated for 4 weeks and disease severity was determined according to Disease Index (DI) [68].

### Mycotoxin analysis

The DON extraction was performed as previously described [69]. For DON analysis in flowering wheat, a10 μL of conidial suspension (1 × 10^6^ conidia/mL) was injected into the center spikelet of a wheat head. Four to five spikes were inoculated per plant. The inoculated spikes were sampled at 5 dpi and stored at -80°C freezer until DON extraction. The inoculated spikes were pooled and ground into fine powder in liquid nitrogen using a mortar and pestle. For DON induction in rice media, a fresh mycelial plug was inoculated on 1 g of rice substrate for 4 weeks. The wheat or rice culture was harvested, ground, and mixed vigorously with 6 mL of 84% acetonitrile for 30 min. After phase separation, the upper phase was filtered through a 0.45 μm syringe filter. Reverse-phase HPLC on a Prominence HPLC system (Shimadzu, Japan) with a C18 column was used for the analysis with a simple modification of the previous methods [70, 71]. For DON detection, the mobile phase was 10% aqueous acetonitrile (ACN), and the flow rate was 1 mL min^−1^. A gradient elution program was applied as follows: after 10% ACN was maintained for 11 min, it linearly increased to 30% ACN at 12 min. It was linearly decreased from 30% ACN at 12 min to 10% ACN at 18 min. Subsequently, 10% ACN was held for 17 min for re-equilibration of the column before injection of the next sample, giving a total run time of 35 min. The diode-array detection was applied (DON was detected at wavelength 237 nm).

### PG plate assay and exo-polygalacturonase activity

PG plate assay was examined as described previously [72]. Reducing sugars from wild-type and Δ*Fopyc1* mutant culture supernatants induced on MM containing pectin (0.5%) as the sole carbon source were determined by the Nelson–Somogyi method, as described previously for the indicated time periods [37].

### Quantification of fungal biomass by qPCR and gene expression by RT–qPCR

qPCR for quantification of gene expression or of fungal biomass in *Solanum lycopersicum* plants was performed as described previously [73]. Briefly, DNA was extracted from roots of three tomato plants after 3 days of inoculation. Genomic DNA was extracted from the roots ground in liquid nitrogen using the cetyl trimethylammonium bromide method [55] and used for the quantification of fungal biomass. qPCR was carried out with SYBR Green Supermix (Bio-Rad, Hercules, CA, USA) and a 7500 real-time PCR system (Applied Biosystems, Foster City, CA, USA) using the corresponding primers (S3 Table). Cycling conditions were 10 min at 95 °C followed by 40 cycles of 10 s at 95 °C, 10 s at 62 °C, and 20 s at 72 °C. Data were analyzed using the ΔΔCt method [74] by calculating the ratio of the plant housekeeping genes *SlGAPDH* (tomato) [75] versus the Fo4287-specific *SIX1* gene (FOXG_16418) to calculate the fungal burden.

For RT-qPCR, total RNA was prepared using the Easy-Spin Total RNA Extraction Kit (Intron Biotech, Seongnam, Republic of Korea). First-strand cDNA was synthesized from total RNA with SuperScript III reverse transcriptase (Invitrogen, Carlsbad, CA, USA). RT-qPCR was carried out using the methods described above. The elongation factor 1-alpha (*EF*) was used as a reference gene for *F*. *graminearum* and actin (*ACT*) for *F*. *oxysporum*. The PCR was repeated three times with three biological replicates. The relative transcript levels of target genes were calculated as previously described [33].

For investigating the *FgTRI5* and *FgTRI6* gene expression in flowering wheat, conidial suspension was infected into the five to six spikelets of a wheat head. The inoculated spikelets were sampled at 5 dpi and stored at -80°C freezer until RNA isolation. The inoculated spikes were pooled and ground into fine powder in liquid nitrogen using a mortar and pestle. Total RNA was isolated as described above.

### Penetration assay

Autoclaved cellophane membrane (Goma Biotech, Seoul, Republic of Korea) and nitrocellulose membrane (GVS, white disk diam. 47 mm, hydrophilic) were placed upon complete medium agar in 60 × 15 mm of petri-dish. 1 mm^2^ agar block was placed on the membrane and incubated for 36 hours for *F*. *graminearum* and 72 hours for *F*. *oxysporum*. Following incubation, the membrane with the agar block on it was removed, and the plate was incubated for 2 days for readout.

### Metabolic profiling of mycelia samples

For mycelia preparation, 5 × 10^6^ conidia of Z-3639, Δ*Fgpyc1*, Fo4287, and Δ*Fopyc1* were grown in CM medium for 24 hours. The mycelia were obtained and washed three times with distilled water. Washed mycelia were transferred to 25 mL MM containing no carbon source in a 100 mL flask, subcultured for 1 hours, and supplemented with 2% (w/v) glucose. Cultures were maintained in a rotary shaker at 150 rpm, 25 °C for the indicated time periods (24 hours and 48 hours). One flask was regarded as one biological replicate. The mycelia were collected on filter paper and washed twice with PBS buffer. Harvested mycelia were freeze-dried for 12 hours, ground with a toothpick, and their masses were unified to 200 mg. Each mycelium milled at 28 Hz for 90 seconds in a 2 mL Eppendorf tube was used for the extraction with 1,400 μL of the extraction solvent (methanol: isopropanol: water, 3:3:2, v/v/v). The mixture was sonicated for 10 min and then centrifuged at 13,200 rpm for 10 min at 4 °C. Each supernatant (650 μL) was transferred and completely dried in a speed vacuum concentrator (SCANVAC, Republic of Korea). The dry extracts were reconstituted with 70% acetonitrile 50 μL and liquid chromatography-orbitrap mass spectrometry analysis.

Waters Acquity UPLC BEH C18 column (2.1 × 100 mm, 1.7 μm particle diameter) was used with 0.1% formic acid in acetonitrile (organic phase) and 0.1% formic acid in water (aqueous phase) as solvents at a flow rate of 0.3 mL/min. A solvent gradient scheme was used, starting at 0.5% organic for 0.1 min, followed by a linear increase to 99.5% organic over 10 min, holding at 99.5% organic for 2 min, decreasing back, and holding at 0.5% organic for 3 min, for a total of 15 min.

### Untargeted analysis using LC-orbitrap MS

LC−MS was performed on a Thermo Fisher Scientific Ultimate 3000 UHPLC system coupled with a Thermo Q-Exactive Plus instrument orbitrap high-resolution mass spectrometer for detection. Mass spectrometer parameters were used as spray voltage 3.8 kV for positive ion mode and 3.5 kV for negative ion mode, capillary temperature 320 °C, probe heater temperature 300 °C; 40 sheath flow rate, 10 auxiliary flow rate, and one spare gas; FullMS scan for resolution 70,000, Top5 MS1 ions; ddMS2 scan mode for resolution 17 500 at 100 m/z; AGC target, 1e5; maximum IT, 50 ms; isolation window, 1.0 m/z; normalized collision energy (NCE), 30, 40, 50; intensity threshold, 2e3 ions; apex trigger, 3–6 s; dynamic exclusion, 6 s. The instrument was calibrated weekly with positive and negative ion calibration solutions (Thermo Fisher Scientific, San José, CA, USA). Each sample was analyzed in negative and positive ionization modes using an m/z range of 80 to 1200.

Data acquisition and pre-processing were conducted using Xcalibur software (Thermo Fisher Scientific, Waltham, MA, USA). LC−MS RAW files were converted to abf format using abfconverter (AnalysisBaseFileConverter), followed by analysis using MS-DIAL ver.4.9.221218 [76] for alignment and identification (MS1 tolerance, 0.005 Da; MS2, 0.05 Da, similarity score: 70%). The data processing was done following the workflow, such as a total score, signal to noise, retention times, and fill percentage. The mass tolerance of MS1 on every node was set at 5 ppm. Align Retention Time node was set to 0.5 min for the maximum shift.

### 3-Nitrophenylhydrazine derivatization for targeted analysis using LC-orbitrap MS

The supernatant (40 μL) of extraction solvent (methanol: isopropanol: water, 3:3:2, v/v/v) was mixed with 20 μL of 200 mM 3-nitrophenylhydrazine-HCL in acetonitrile (70%) and 20 μL of a 1-ethyl-3-(3-dimethylaminopropyl) carbodiimide-HCL (120 mM) dissolved in 6% pyridine solution. The mixture was incubated for 30 min at 40 °C. A 2 μL aliquot of the reaction mixture was analyzed by LC/MS using the gradient scheme described above. Mass spectra were acquired using Q-Exactive Plus Orbitrap (Thermo Fisher Scientific, Waltham, MA, USA) equipped with an electrospray ionization interface (HESI-II) in negative ionization, and the system was controlled using Xcalibur 4.0 and Q-Exactive Tune software. Raw data were processed by Tracefinder software (version 4.1, Thermo Fisher Scientific, San José, CA, USA). Mass tolerance for precursor ion and retention time tolerance were set to 5 ppm and 0.5 min, respectively.

The same procedure was performed for a metabolome fraction containing OAA. Standards of OAA were derivatized with 3-NPH as described above. The 3-NPH derivatives of OAA derived from three other forms showed different retention times.

### Data analysis

Statistical analyses were conducted for all continuous variables. Missing value imputations were filled by 20% of the group minimum. Univariate statistics and multiple linear regression [77] (MLR) were performed using R. Principle component analysis (PCA) and unique structure plots were performed using SIMCA 17 software (Sartorius, Göttingen, Germany). Pathway analysis and pattern searching modules were performed using MetaboAnalyst 5.0 [78]. Mapping of metabolites by chemical and biochemical relation network was created using Metamapp [79] and visualized in Cytoscape (3.10.0).

## Supporting information

S1 Table Metabolomic profiling data. (CSV)

S2 Table Multiple linear regression data. (CSV)

S3 Table Primers used in this study. (DOCX)

S1 Fig Characterization of the pyruvate carboxylase enzyme *Fg*Pyc1. A) Domain architecture of *Fg*Pyc1. Conserved domains were identified by InterPro analysis. Three functional domains including biotin carboxylation (BC) domain, carboxyltransferase (CT) domain, and biotin carrier (BCCP) domain were conserved in *Fg*Pyc1, where XP_011326775.1 contains C2H2-zinc finger (Znf) motif and FGRAMPH1_01T23899 contains only two domains, BC and CT. B) Phylogenetic tree of Pyc1 orthologs. Amino acid sequences were aligned using the ClustalW, and RaxmL was used to perform phylogenetic analysis using the maximum likelihood method with 1000 bootstrap replicates.
(TIF)

S2 Fig Deletion of *PYC1* gene in *F*. *graminearum* and *F*. *oxysporum*. A) Deletion and complementation of *FgPYC1*. Lane 1, wild-type strain Z-3639; lane 2 and 3, *FgPYC1* deletion mutant strains; lane 4 and 5, *FgPYC1* deletion mutant-derived strain complemented with *FgPYC1-GFP*. The sizes of DNA standards (kb) are indicated on the left of each blot. B) Deletion of *FoPYC1*. Lane 1, wild-type strain Fo4287; land 2 and 3, *FoPYC1* deletion mutant strains. H, HindIII; S, SmaI; *GEN*, geneticin resistance gene cassette; *HYG*, hygromycin B resistance gene cassette. The sizes of DNA standards (kb) are indicated on the left of each blot.
(TIF)

S3 Fig DON analysis in *PYC1* deletion mutant strains. A) Transcript levels of DON biosynthetic genes in *F*. *graminearum* strains relative to actin were measured by quantitative reverse transcription-polymerase chain reaction (qRT-PCR). Conidial suspensions were inoculated into center spikelets in the flowering wheat head. The inoculated spikelets were sampled at 5 dpi. The relative transcript abundances of the indicated gene in Z-3639 were arbitrarily set to 1. B) Deoxynivalenol (DON) production. A fresh mycelial plug was inoculated on 1 g of rice substrate for four weeks. The rice samples were ground and extracted with 84% acetonitrile. Reverse-phase HPLC with a C18 column was used for the analysis for DON detection.
(TIF)

S4 Fig Interchangeability of *PYC1* between *F*. *graminearum* and *F*. *oxysporum*. A) Mycelial growth of Δ*Fopyc1* · FgPYC1oe mutant strains on CM and MM. Pictures were taken 7 days after inoculation. B) Fusarium wilt disease assay on tomatoes with *F*. *oxysporum* strains. Two-week old seedlings were inoculated using a dipping protocol with 5 × 10^6^ microconidia mL^-1^ or water (mock inoculation). The pictures were taken 28 days after inoculation.
(TIF)

S5 Fig Mycelial growth of *PYC1* deletion mutants under aspartate and OAA supplementation. Mycelial growth of A) *F*. *graminearum* and B) *F*. *oxysporum* strains on minimal medium (MM) with 5 mM aspartate and 5 mM OAA, respectively. Pictures were taken 3 days after inoculation.
(TIF)

S6 Fig Fusarium wilt assay with *F*. *oxysporum* strains supplemented with aspartate. A) Phytotoxicity of aspartate in tomato roots. Aspartate solution with pH adjusted to 7.0 was applied to the soil every two days. Images were captured two weeks after watering. B) Fusarium wilt disease assay on tomatoes with *F*. *oxysporum* strains. Two-week old seedlings were inoculated using a dipping protocol with 5 × 10^6^ microconidia mL^-1^ or water (mock inoculation). After root dipping, 5 mM aspartate solution was applied to the soil every two days. The pictures were taken 28 days after inoculation.
(TIF)

S7 Fig Target peak extracted ion chromatogram of oxaloacetate standard compound. (TIF)

S8 Fig Detection of oxaloacetate 3-NPH derivatization in target metabolic analysis. (TIF)

S9 Fig Detection of pyruvate 3-NPH derivatization in target metabolic analysis. (TIF)

S10 Fig Reconstructed metabolic network based on chemical similarity and KEGG reaction pair (24 h culture). Red and blue color represent the metabolites that are significantly up- and down-regulation in Δ*pyc1* mutants compared to the wild-type strain.
(TIF)
