# Supplementary material for: Oxaloacetate anaplerosis differently contributes to pathogenicity in plant pathogenic fungi Fusarium graminearum and F. oxysporum
Source: PLoS Pathog. 2024 Sep 9;20(9):e1012544. doi: 10.1371/journal.ppat.1012544 (PMC11412510; doi:10.1371/journal.ppat.1012544)
Supplement: S3 Table — (DOCX) [file ppat.1012544.s003.docx]

**S3 Table.** Primers used in this study.

| **Primer** | **Sequence (5'→3')** | **Description** |
| --- | --- | --- |
| FgPC-RACE-3R | GATTACGCCAAGCTTCCGGGCGGAAGGAATGCATGGGTAGTC | For RACE-PCR of *FgPYC1* |
| FgPC/5F | CGATTTGTTGAGGAGGGGATTTCT | Forward and reverse primers for amplification of 5’ flanking region of *FgPYC1* with tail for the geneticin resistance gene cassette fusion |
| FgPC/5R | gcacaggtacacttgtttagagCGATTTACTTCAGTGGTTTCGTCAGAG |  |
| FgPC/3F | ccttcaatatcatcttctgtcgCGTGACGAAAGCGCCCTAATC | Forward and reverse primers for amplification of 3’ flanking region of *FgPYC1* with tail for the geneticin resistance gene cassette fusion |
| FgPC/3R | CATTGCATCTCCTTCTCAACTCGTC |  |
| FgPC/5N | AAACAAAGATGTGTCGGGGGTATGAG | Forward and reverse nest primers for third fusion PCR for amplification of *FgPYC1* deletion construct |
| FgPC/3N | AACGCCATGACTTGTCGCCACT |  |
| FgPC/with 5F | AGCCTGTGGGTGGTTAGCGTC | For co-dominant PCR of *FgPYC1* |
| FoPC-5F | TCGGCGGGTGTTGAGATTATGT | Forward and reverse primers for amplification of 5’ flanking region of *FoPYC1* with tail for the geneticin resistance gene cassette fusion |
| FoPC-5R | gcacaggtacacttgtttagagGGCGATCTGTGTCAGCGACTTC |  |
| FoPC-3F | ccttcaatatcatcttctgtcgTGCGCAAGGAAATAAAAGATTGGG | Forward and reverse primers for amplification of 3’ flanking region of *FoPYC1* with tail for the geneticin resistance gene cassette fusion |
| FoPC-3R | GTCTGCCCTATCGCCTGGTCTTAC |  |
| FoPC-5N | ATACTCCCCCATGCCTGTGTGAC | Forward and reverse nest primers for third fusion PCR for amplification of *FoPYC1* deletion construct |
| FoPC-3N | GCGGAGTATATTCAAGATTCGTCCC |  |
| FoPC with 5F | GGAAACGCTCAACGAAGACAGTTC | For co-dominant PCR of *FoPYC* |
| Gen-for | CGACAGAAGATGATATTGAAGG | Forward and reverse primers for amplification of the geneticin cassette from the pII99 vector |
| Gen-rev | CTCTAAACAAGTGTACCTGTG |  |
| FoPC com-seq1 | CAAAGATGTGTCGGCGGAATG | Forward and reverse primers for pDL2-*FoPYC* plasmid identification |
| FoPC com-seq2 | TGCCCTCATTGCTTCTTCCATTT |  |
| FoPC com-seq3 | ATACCGACAAGCGTGCTCATTACC |  |
| FoPC com-seq4 | CAAGCTGACACGGATTTCTGGTT |  |
| FoPC com-seq5 | GCTGTTGAGAGGTACGAGGAGGTC |  |
| FoPC com-seq6 | CTTTCCTTGCCTCCCTCCTTACT |  |
| FoPC com-seq7 | GGTGTCGATATCTTCCGTGTCTTT |  |
| FoPC com-seq8 | CCTTGAGGGCAGCGAGTGTG |  |
| FoPC com-seq9 | CGCGCCTATGTCTGGTGTCCT |  |
| FoPC com-seq10 | ACCATCAACAGAATCACCCTCACG |  |
| FoPC com-seq11 | GGTCAGGGTACTTCTTTCGGATTG |  |
| FoPC com-seq12 | TTGGTGCAGGGTGTGGAAGT |  |
| FoPC com-seq13 | CTCGATCTTCCCAGTGTCAGGTG |  |
| FoPC com-seq14 | TGTCAGTGCCATAGTTCAGGTCAT |  |
| FoPC com-seq15 | ATCGTTGAATGTGAGTGGTTGCTG |  |
| FoPC com-seq16 | CAGGACATGCCACTAAAACACGAT |  |
| FoPC com-seq17 | CGAAACGCGACGATTGACA |  |
| FoPC-F com | TATAGGGCGAATTGGGTACTCAAATTGGTTTCGGCGGGTGTTGAGATTATGT | Forward and reverse primers for amplification of *Fo*Pyc open reading frame for GFP fusion protein construction under native promoter |
| FoPC-R com | CCCGGTGAACAGCTCCTCGCCCTTGCTCACGGCCTTCTCGATTCGACACAC |  |
| pg1-1 | GTCACTTCGGGTACAAACATC | For real-time PCR of *PG1* |
| pg1-2 | CCTTGATGAACTTGATGCCGC |  |
| pgx4-1 | GTACAGCATTGCCTCGCCAC | For real-time PCR of *PGX4* |
| pgx4-2 | CGGGTTTCTCATTCGCAGGTT |  |
| pg5-1 | GCCTGGTCGCCTCCGTACT | For real-time PCR of *PG5* |
| pg5-2 | TCTTCTTGCCGCCGTTGCTGCCCTTGCCGT |  |
| pgx6-1 | GAAGTCATCGCAAGGTCTATAC | For real-time PCR of *PGX6* |
| pgx6-2 | AGAACAGAATAGGTCGGAGGTA |  |
| act-q7 | ATGTCACCACCTTCAACTCCA | For real-time PCR of *ACT* |
| act-q8 | CTCTCGTCGTACTCCTGCTT |  |
| chsV-3 | ACAGCTCCAACGAACTCTCTT | For real-time PCR of *CHSV* |
| chsV-26 | GGAGGTACTTGGTCATGTCGT |  |
| gadph-1 | TGATTTGAACTCGTCGCAG | For real-time PCR of *SIGAPDH* |
| gadph-2 | CCAAAAACAGTAACAGTAACAGCCTTC |  |
| six-1-1 | ATAGCATGGTACTCCTTGGCG | For real-time PCR of *SIX1* |
| six-1-2 | CCTGATGGTGACGGTTACGAA |  |
