# Supplementary figures and images for: Oxaloacetate anaplerosis differently contributes to pathogenicity in plant pathogenic fungi Fusarium graminearum and F. oxysporum
Source: PLoS Pathog. 2024 Sep 9;20(9):e1012544. doi: 10.1371/journal.ppat.1012544 (PMC11412510; doi:10.1371/journal.ppat.1012544)

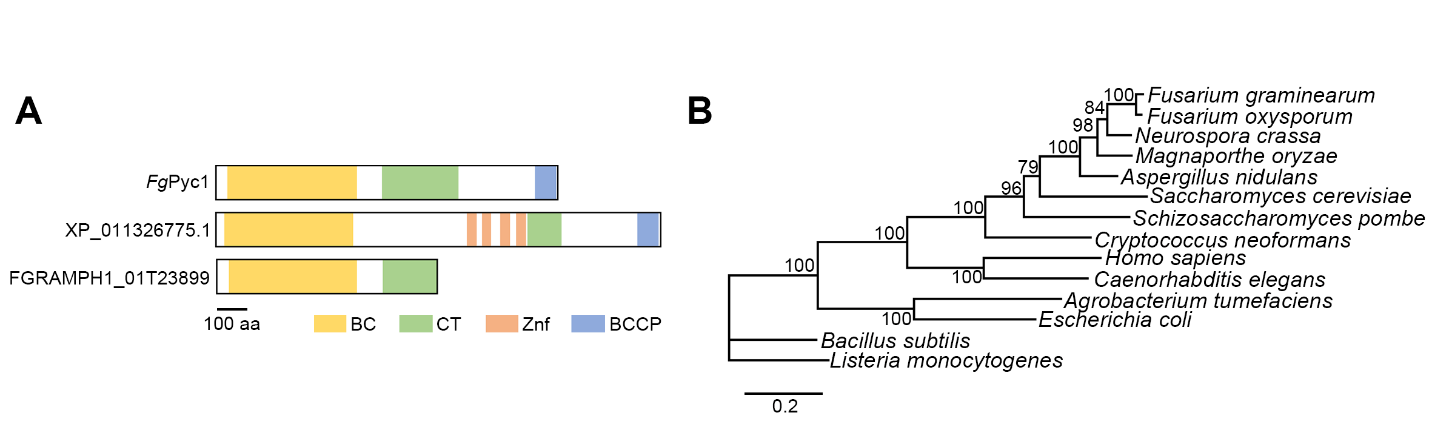

Supplement: S1 Fig — A) Domain architecture of FgPyc1. Conserved domains were identified by InterPro analysis. Three functional domains including biotin carboxylation (BC) domain, carboxyltransferase (CT) domain, and biotin carrier (BCCP) domain were conserved in FgPyc1, where XP_011326775.1 contains C2H2-zinc finger (Znf) motif and FGRAMPH1_01T23899 contains only two domains, BC and CT. B) Phylogenetic tree of Pyc1 orthologs. Amino acid sequences were aligned using the ClustalW, and RaxmL was used to perform phylogenetic analysis using the maximum likelihood method with 1000 bootstrap replicates. (TIF) [file ppat.1012544.s004.tif]

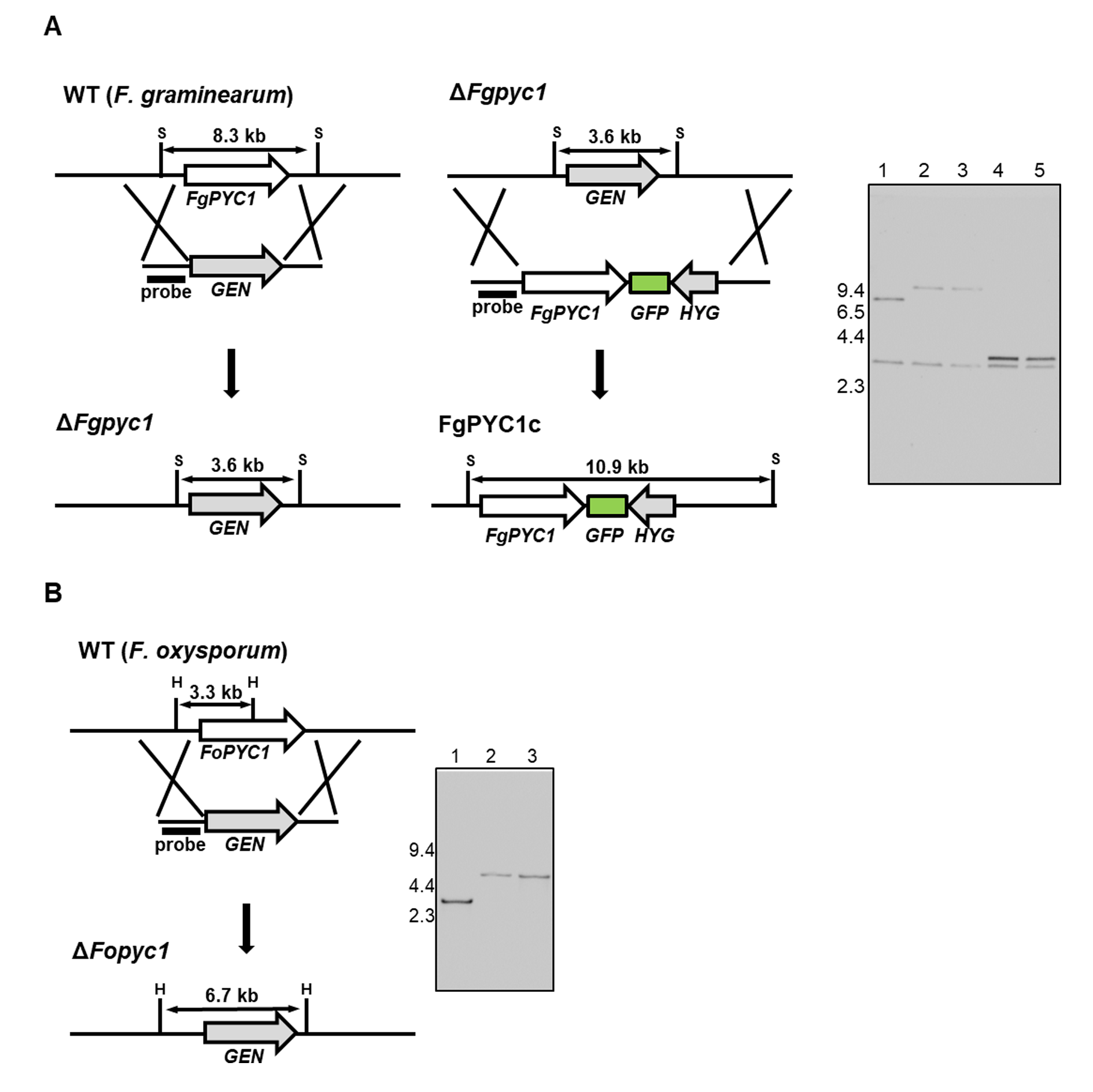

Supplement: S2 Fig — A) Deletion and complementation of FgPYC1. Lane 1, wild-type strain Z-3639; lane 2 and 3, FgPYC1 deletion mutant strains; lane 4 and 5, FgPYC1 deletion mutant-derived strain complemented with FgPYC1-GFP. The sizes of DNA standards (kb) are indicated on the left of each blot. B) Deletion of FoPYC1. Lane 1, wild-type strain Fo4287; land 2 and 3, FoPYC1 deletion mutant strains. H, HindIII; S, SmaI; GEN, geneticin resistance gene cassette; HYG, hygromycin B resistance gene cassette. The sizes of DNA standards (kb) are indicated on the left of each blot. (TIF) [file ppat.1012544.s005.tif]

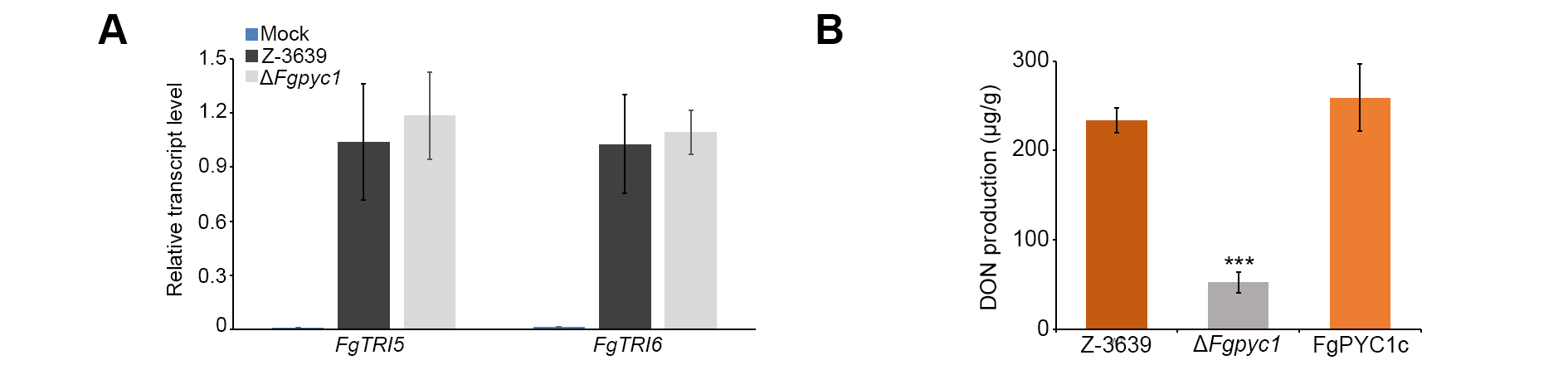

Supplement: S3 Fig — A) Transcript levels of DON biosynthetic genes in F. graminearum strains relative to actin were measured by quantitative reverse transcription-polymerase chain reaction (qRT-PCR). Conidial suspensions were inoculated into center spikelets in the flowering wheat head. The inoculated spikelets were sampled at 5 dpi. The relative transcript abundances of the indicated gene in Z-3639 were arbitrarily set to 1. B) Deoxynivalenol (DON) production. A fresh mycelial plug was inoculated on 1 g of rice substrate for four weeks. The rice samples were ground and extracted with 84% acetonitrile. Reverse-phase HPLC with a C18 column was used for the analysis for DON detection. (TIF) [file ppat.1012544.s006.tif]

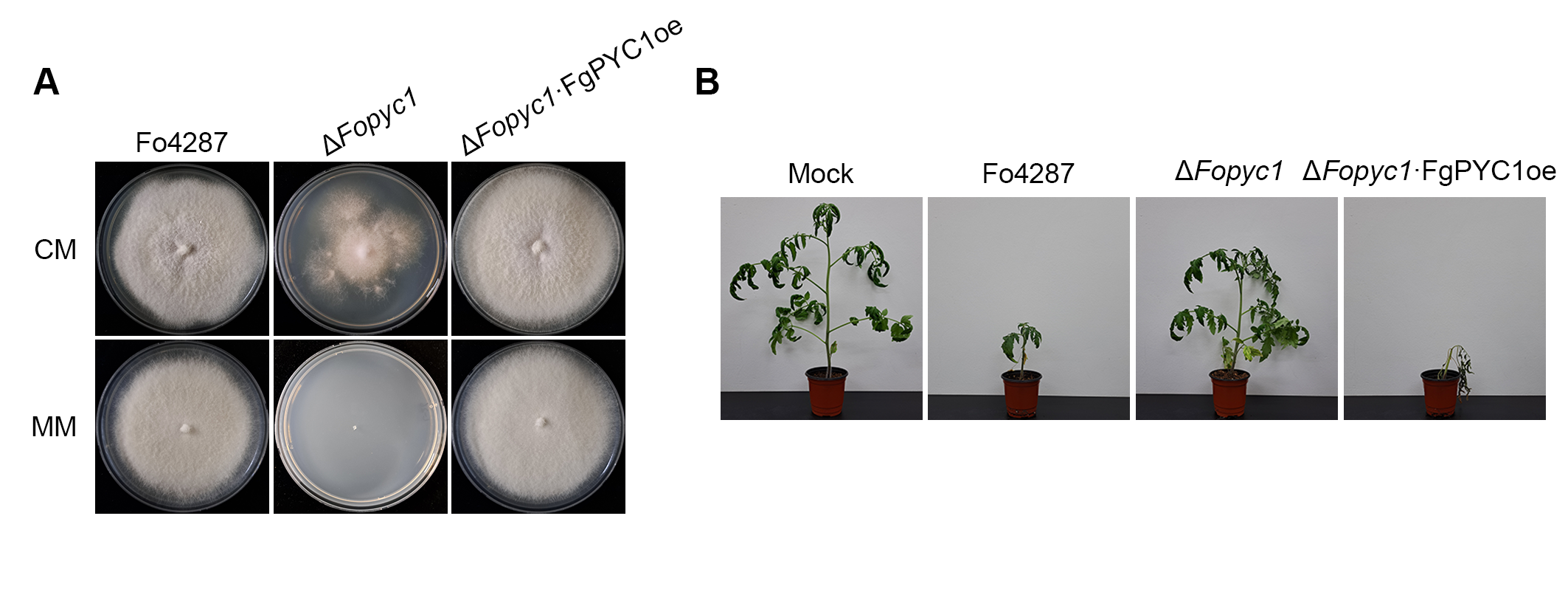

Supplement: S4 Fig — A) Mycelial growth of ΔFopyc1 · FgPYC1oe mutant strains on CM and MM. Pictures were taken 7 days after inoculation. B) Fusarium wilt disease assay on tomatoes with F. oxysporum strains. Two-week old seedlings were inoculated using a dipping protocol with 5 × 10^6^ microconidia mL-1 or water (mock inoculation). The pictures were taken 28 days after inoculation. (TIF) [file ppat.1012544.s007.tif]

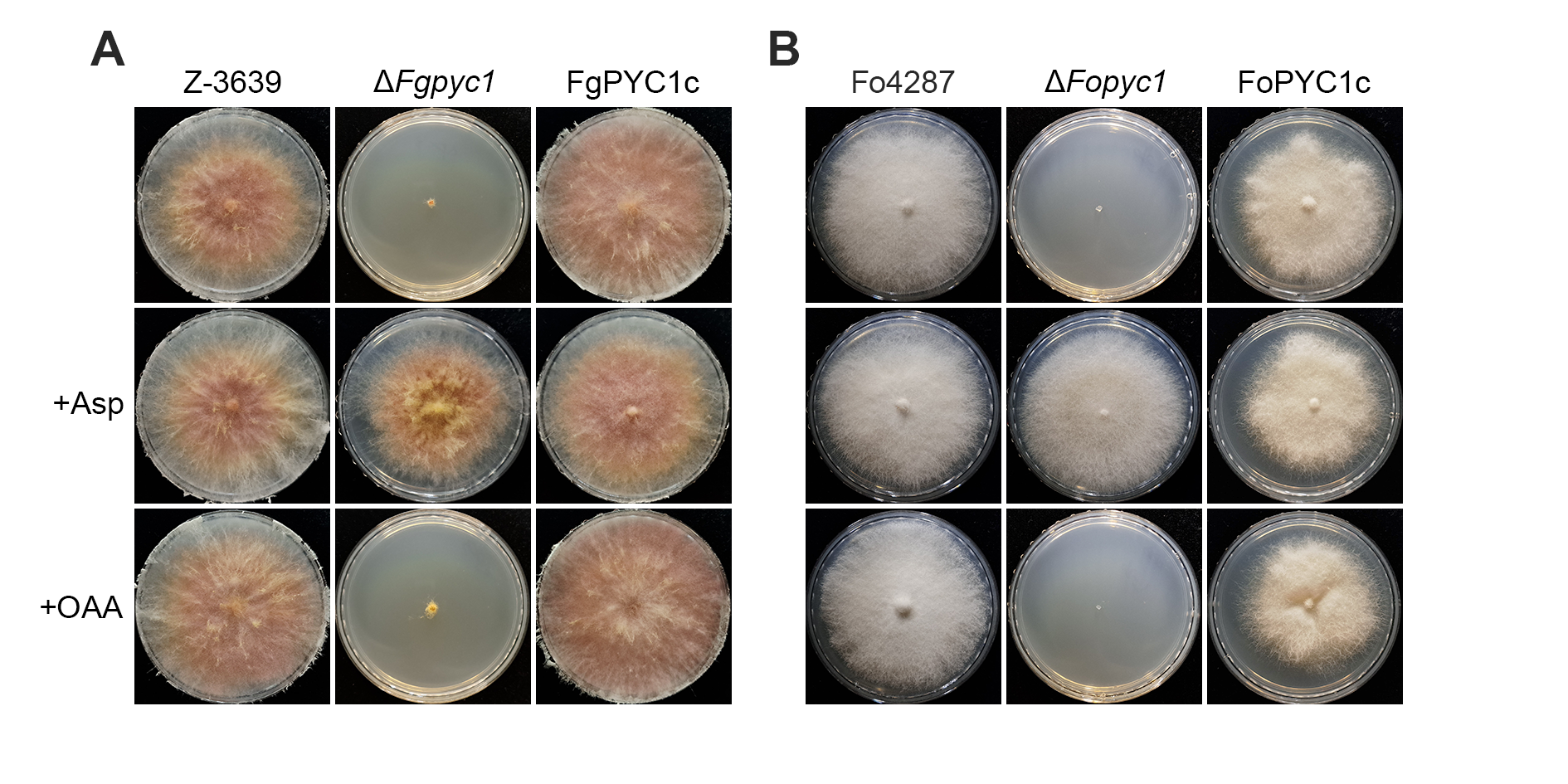

Supplement: S5 Fig — Mycelial growth of A) F. graminearum and B) F. oxysporum strains on minimal medium (MM) with 5 mM aspartate and 5 mM OAA, respectively. Pictures were taken 3 days after inoculation. (TIF) [file ppat.1012544.s008.tif]

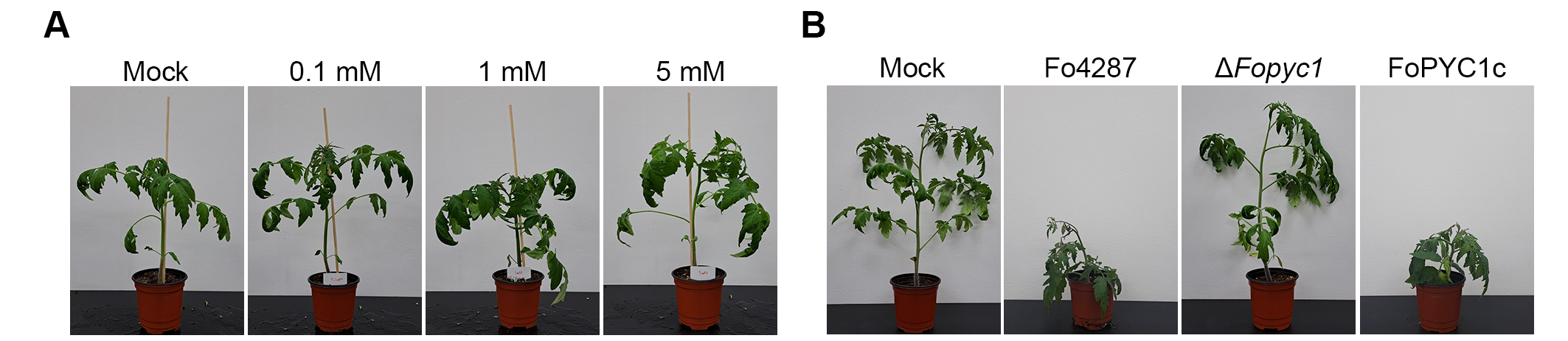

Supplement: S6 Fig — A) Phytotoxicity of aspartate in tomato roots. Aspartate solution with pH adjusted to 7.0 was applied to the soil every two days. Images were captured two weeks after watering. B) Fusarium wilt disease assay on tomatoes with F. oxysporum strains. Two-week old seedlings were inoculated using a dipping protocol with 5 × 10^6^ microconidia mL-1 or water (mock inoculation). After root dipping, 5 mM aspartate solution was applied to the soil every two days. The pictures were taken 28 days after inoculation. (TIF) [file ppat.1012544.s009.tif]

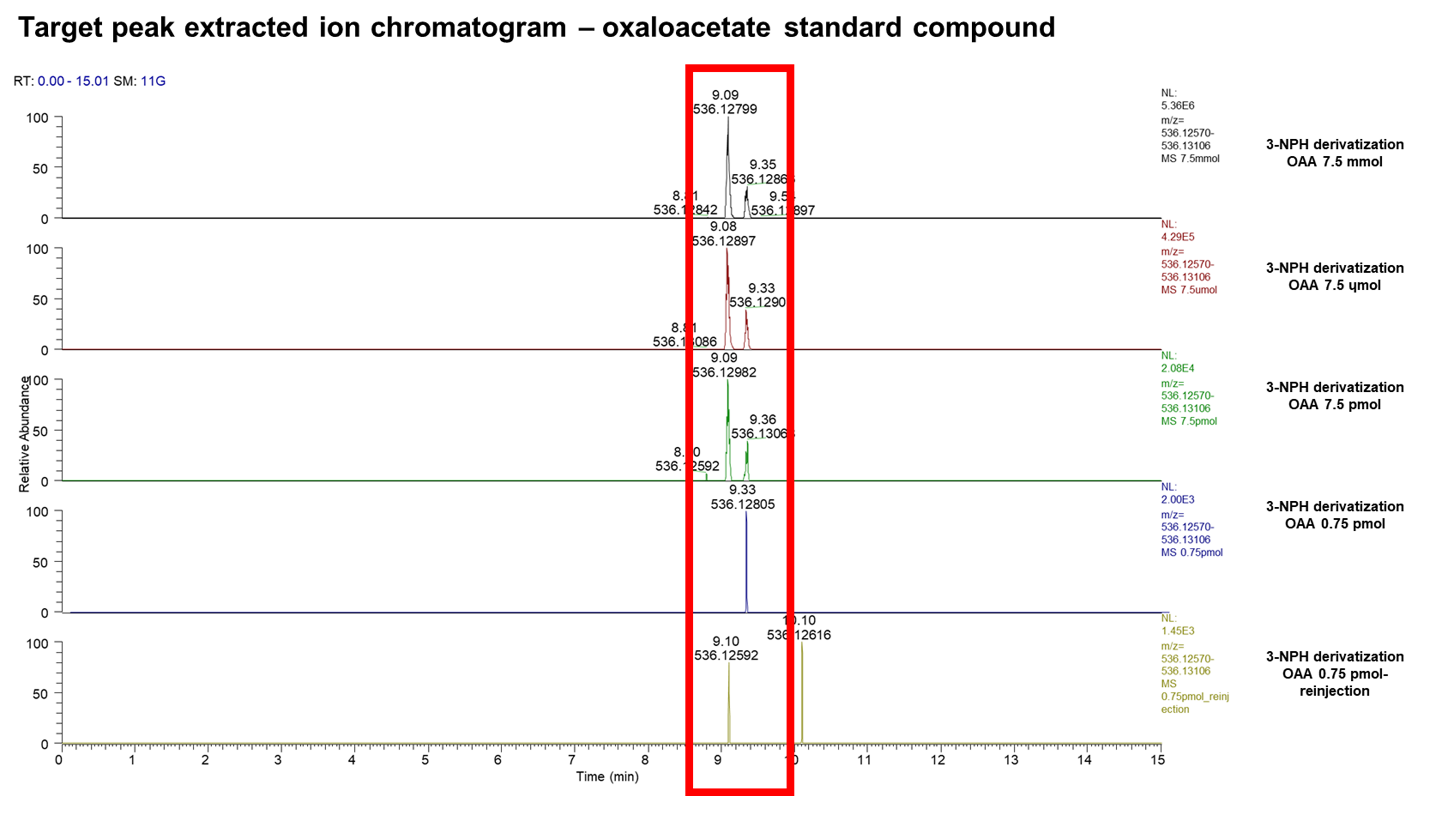

Supplement: S7 Fig — (TIF) [file ppat.1012544.s010.tif]

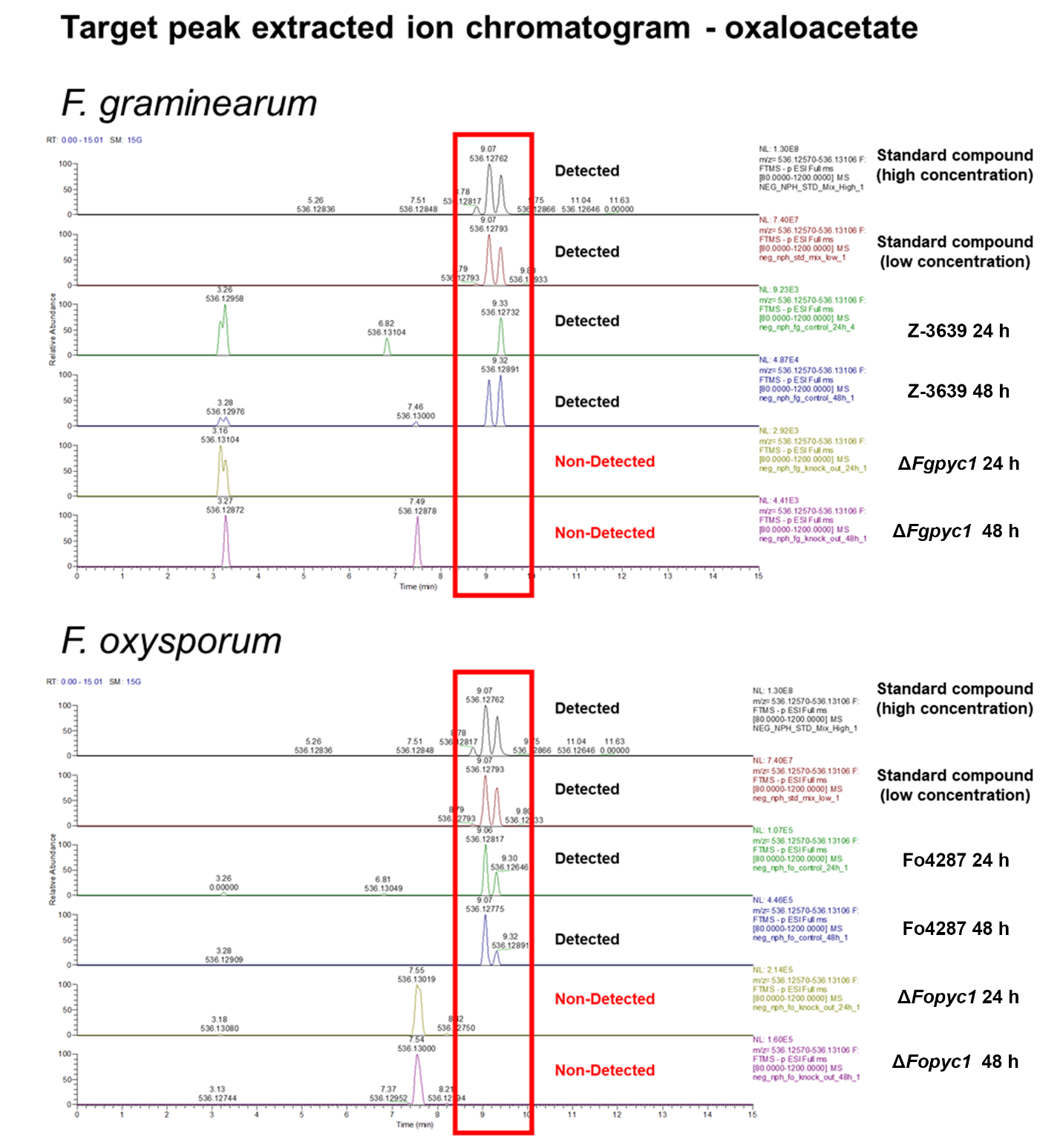

Supplement: S8 Fig — (TIF) [file ppat.1012544.s011.tif]

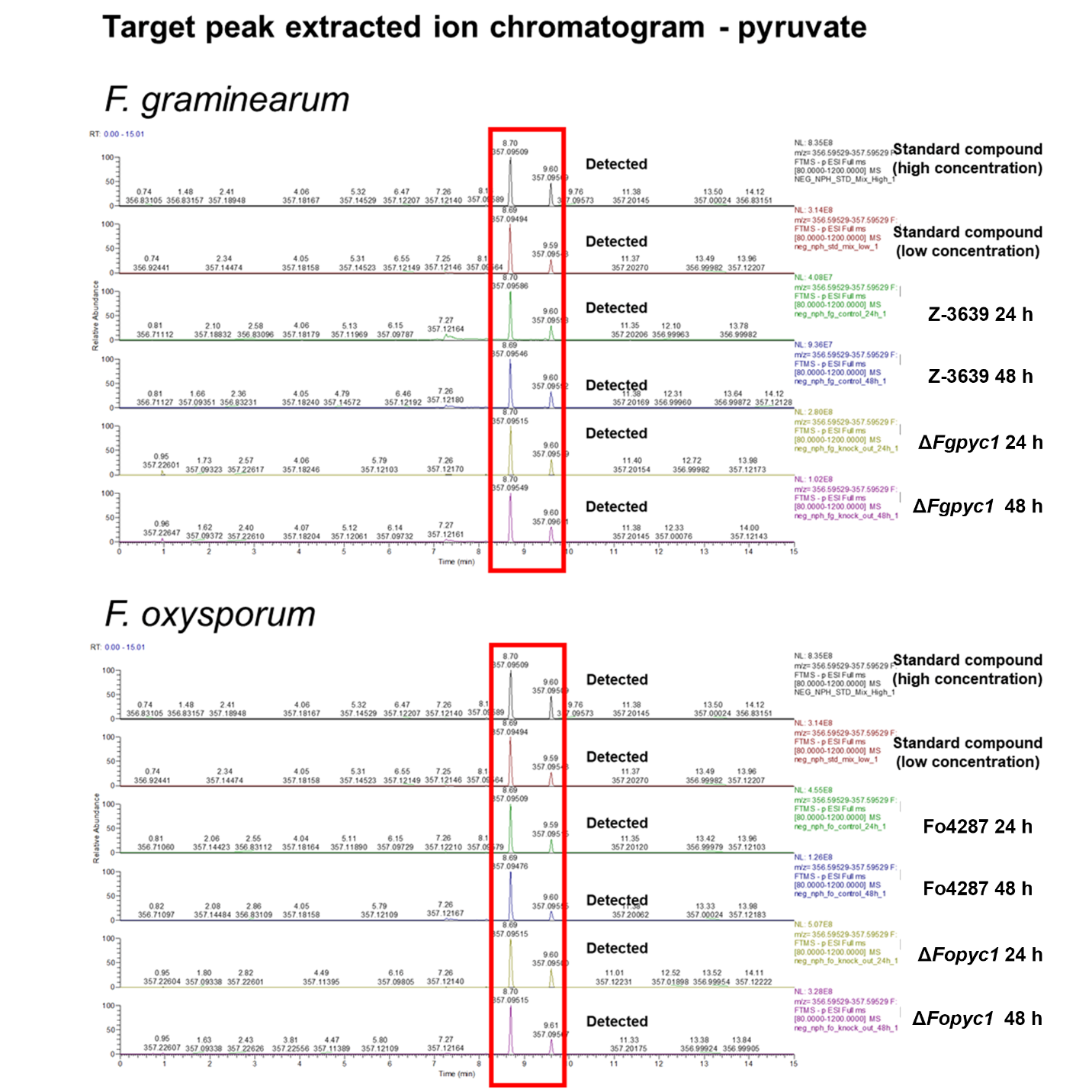

Supplement: S9 Fig — (TIF) [file ppat.1012544.s012.tif]

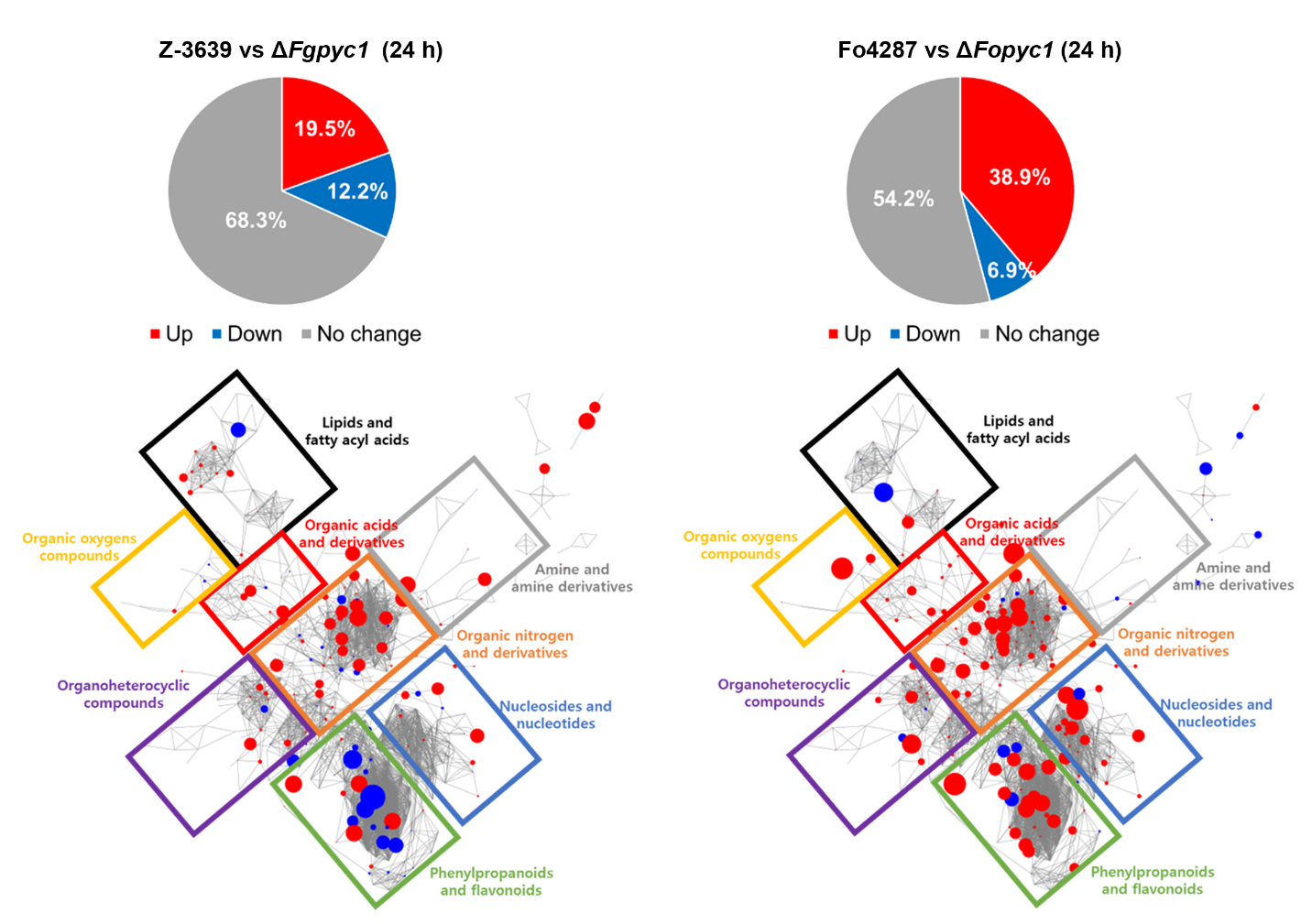

Supplement: S10 Fig — Red and blue color represent the metabolites that are significantly up- and down-regulation in Δpyc1 mutants compared to the wild-type strain. (TIF) [file ppat.1012544.s013.tif]
